# Deep Learning‐Based Ion Channel Kinetics Analysis for Automated Patch Clamp Recording

**DOI:** 10.1002/advs.202404166

**Published:** 2024-12-31

**Authors:** Shengjie Yang, Jiaqi Xue, Ziqi Li, Shiqing Zhang, Zhang Zhang, Zhifeng Huang, Ken Kin Lam Yung, King Wai Chiu Lai

**Affiliations:** ^1^ Department of Biomedical Engineering City University of Hong Kong Tat Chee Avenue, Kowloon Tong Kowloon Hong Kong SAR China; ^2^ JNU‐HKUST Joint Laboratory for Neuroscience and Innovative Drug Research College of Pharmacy Jinan University 601 West Huangpu Road, Tianhe Guangzhou 510632 China; ^3^ School of Public Health Guangzhou Medical University Xinzao, Panyu Guangzhou 511436 China; ^4^ Department of Chemistry Chinese University of Hong Kong Shatin New Territories Hong Kong SAR China; ^5^ Department of Science and Environmental Studies Education University of Hong Kong 10 Lo Ping Road Tai Po New Territories Hong Kong SAR China

**Keywords:** deep learning, electrophysiology, ion channels, patch clamp, whole‐cell recording

## Abstract

The patch clamp technique is a fundamental tool for investigating ion channel dynamics and electrophysiological properties. This study proposes the first artificial intelligence framework for characterizing multiple ion channel kinetics of whole‐cell recordings. The framework integrates machine learning for anomaly detection and deep learning for multi‐class classification. The anomaly detection excludes recordings that are incompatible with ion channel behavior. The multi‐class classification combined a 1D convolutional neural network, bidirectional long short‐term memory, and an attention mechanism to capture the spatiotemporal patterns of the recordings. The framework achieves an accuracy of 97.58% in classifying 124 test datasets into six categories based on ion channel kinetics. The utility of the novel framework is demonstrated in two applications: Alzheimer's disease drug screening and nanomatrix‐induced neuronal differentiation. In drug screening, the framework illustrates the inhibitory effects of memantine on endogenous channels, and antagonistic interactions among potassium, magnesium, and calcium ion channels. For nanomatrix‐induced differentiation, the classifier indicates the effects of differentiation conditions on sodium and potassium channels associated with action potentials, validating the functional properties of differentiated neurons for Parkinson's disease treatment. The proposed framework is promising for enhancing the efficiency and accuracy of ion channel kinetics analysis in electrophysiological research.

## Graphical abstract

The study introduces an AI framework for analyzing ion channel kinetics in whole‐cell recordings, integrating machine learning for anomaly detection and deep learning for multi‐class classification. Achieving 97.58% accuracy after validation, the framework is applied to drug screening for Alzheimer's disease and nanomatrix‐induced neuronal differentiation. This novel approach promises enhanced efficiency and accuracy in electrophysiological research.

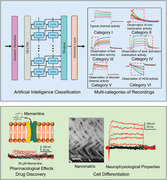

## 1 Introduction

The patch clamp technique has long been indispensable in electrophysiological research. Patch clamp recordings play a central role in elucidating mechanisms underlying various pharmacological, physiological, and biophysical phenomena.^[^ ^1^, ^2^, ^3^, ^4^ ^]^ However, patch clamp applications face significant challenges in terms of recording acquisition and analysis.^[^ ^5^, ^6^ ^]^ For example, in cutting‐edge protein structure prediction involving patch clamp, the classification of protein characteristics requires skilled personnel to perform labor‐intensive and time‐consuming operations.^[^ ^7^, ^8^ ^]^ Automated processing can be advantageous for manual identification and can accelerate progress in ion channel research, drug discovery, and disease modeling. Therefore, it is critical to develop automated methods to analyze ion channel kinetics from patch clamp recordings.

Whole‐cell recording is the most commonly used patch clamp method for providing an integrated view of ion channel activity, including synaptic transmission, action potential generation, pharmacological testing, and intracellular signaling.^[^ ^9^, ^10^ ^]^ Ion channel kinetics analysis of whole‐cell recording is a fundamental tool for investigating cellular physiological states by classifying dynamic properties and identifying underlying mechanisms.^[^ ^11^ ^]^ For instance, understanding the kinetic properties of ion channels in different types of neurons enhances comprehension of neural circuit function and brain information processing.^[^ ^12^ ^]^ Studying ion channel kinetics in disease models can reveal how altered ion channel function (e.g., activation, conductance, reversal potential) contributes to disease pathogenesis and progression, as well as the action of potential therapeutic agents.^[^ ^12^, ^13^ ^]^ Artificial intelligence has advanced the analysis of patch clamp recording.^[^ ^14^, ^15^, ^16^, ^17^ ^]^ However, existing deep learning methods are primarily designed to distinguish gating actions of single‐channel recording.^[^ ^18^, ^19^ ^]^ Analysis of single‐channel recordings involves measuring the specific openings and closings of individual channels and quantifying the current passing through a single channel when it is open.^[^ ^20^ ^]^ The implementation can automatically idealize complex ion channel activity to capture the movement of individual proteins and identify gating dynamics in different cell types.^[^ ^18^, ^19^, ^20^ ^]^ Those methods focus on specific ion channels' selectivity and gating behaviors. While artificial intelligence has facilitated the identification of gating actions, there is a critical need for ion channel kinetics analysis of whole‐cell recordings to advance research in diverse fields, including clinical pathology, drug discovery, and neuroscience.

In general, the ion channel kinetics analysis of patch clamp recording signals can start with a multi‐class classification. Classification based on the activity of multiple ion channels has substantial practical significance for recordings originating from diverse testing conditions, such as the treatment of neurodegenerative diseases.^[^ ^21^, ^22^, ^23^ ^]^ Alzheimer's disease is a neurodegenerative condition associated with aging. Current treatments primarily target N‐Methyl‐D‐aspartic acid (NMDA) receptors; however, the 99.5% failure rate of these therapeutic strategies has prompted investigations into voltage‐gated potassium and sodium channels, calcium homeostasis, and magnesium ions as potential pathological pathways.^[^ ^24^, ^25^, ^26^ ^]^ Rapid and precise screening of the effects of potential therapeutic drugs on endogenous ion channels is imperative. For drug screening in Alzheimer's disease, the classification of recordings revels ion channel kinetics, such as voltage dependency, activation threshold, and reversal potential. These classification results facilitate the identification of pharmacological effects and provide evidence for potential drug mechanisms. Furthermore, constructing multiclass recordings enables precise identification of cell physiological states.^[^ ^27^ ^]^ Induced cell differentiation can be used to study the underlying mechanisms of various diseases, such as Parkinson's disease and cardiovascular disease.^[^ ^28^, ^29^ ^]^ Classified recordings can be used to validate the effectiveness of cell differentiation protocols by acquiring specific electrophysiological properties. Hence, there is a critical and unmet need in patch clamp research to develop a multi‐class classification method based on the distinct characteristics of different ion channels.

Analyzing ion channel kinetics of whole‐cell recordings requires the exclusion of anomalous recordings and the multi‐class classification of the remaining data. Anomaly detection characterizes the mathematical properties of patch clamp recordings to identify abnormal recordings that significantly deviate from typical ion channel behavior. K‐nearest neighbors (KNN) adapt well to local changes in the recording distribution since it relies on local neighborhood information.^[^ ^30^, ^31^ ^]^ Utilizing the KNN mathematical approach can effectively process high‐dimensional data while maintaining robustness against noisy data points. Identification of ion channel dynamics is fundamental to multi‐class classification, which requires the capture of the spatiotemporal patterns in the recordings.^[^ ^32^, ^33^ ^]^ The 1Dconvolutional neural network (1DCNN) is a robust framework for time‐series data involving multiple variables.^[^ ^34^ ^]^ The convolutional layer slides 1D filters across the input sequence to detect patterns and capture local dependencies. It is particularly effective for extracting spatial features and identifying ion channel dynamics. Bidirectional long short‐term memory (BiLSTM) incorporates information from both the past and future time steps of sequential data.^[^ ^35^ ^]^ BiLSTM effectively captures temporal dependencies in patch‐clamp recordings and elucidates dynamic changes in ion channel activity over time. The attention layer assigns varying weights to each element to produce accurate classifications.^[^ ^36^ ^]^ Models can effectively handle long sequences and complex dependencies by incorporating an attention layer, thereby improving performance. By integrating the 1DCNN, BiLSTM, and Attention mechanisms into a single model, the classifier's ability to detect dynamic ion channel behavior in patch clamp recordings can be improved. Artificial intelligence offers an efficient and reliable approach for classifying cell types and identifying their physiological activities.

In this study, an artificial intelligence framework has been proposed for ion channel kinetics analysis of whole‐cell recordings. Initially, recordings with functional failure ion channels were filtered using KNN‐based anomaly detection. Subsequently, the remaining recordings underwent multi‐class classification using a 1DCNN‐BiLSTM‐Attention mechanism deep learning model. After training on 240 datasets, the 97.22% accuracy of the 144 testing datasets indicated the anomaly detection ability to resolve the activities of the ion channels. For the deep learning model, we demonstrated the model architecture, testing dataset results, and feature discrimination capabilities. The model was trained using 139 datasets and successfully recognized ion channel behaviors in 124 testing datasets across six categories with 97.58% accuracy. This framework can effectively identify recording features and understand the interactions between response currents by segmenting recordings into distinct phases based on ion channel characteristics. We evaluated the multi‐class classification capabilities of our framework in two applications: drug screening for Alzheimer's disease and characterization of nanomatrix‐induced neural stem cells (NSCs) differentiation. Memantine, an antagonist of the NMDA receptor, is used to slow neurotoxicity in Alzheimer's disease. The impact of memantine on endogenous receptors is yet to be fully understood and continues to be an active area of research.^[^ ^26^, ^37^ ^]^ Well‐established whole‐cell recordings under various drug conditions were obtained to facilitate the study of memantine. By employing our framework to classify whole‐cell recordings, we identified the voltage‐dependent inhibitory effects of memantine on endogenous channels and its antagonistic interaction with calcium ions. The drug screening application demonstrates the accuracy and reliability of the proposed artificial intelligence framework. To assess the effectiveness and generalizability of our framework in real‐world applications, we acquired newly unseen whole‐cell recordings from nanomatrix‐induced NSCs differentiation. The nanomatrix promotes the differentiation of dopamine neurons and supports cell therapy in Parkinsonian rats.^[^ ^38^, ^39^ ^]^ The framework identifies the functional signaling properties of differentiated neurons by evaluating peak current density and inward and outward channel dynamics in the newly unseen whole‐cell recordings. Our framework offers a precise, rapid, and automated method for ion channel kinetics analysis, potentially improving the efficiency and accuracy of data analysis in the critical domain of electrophysiological research.

## 2 Artificial Intelligence Framework

### 2.1 Ion Channel and Recording Classification

**Figure 1:**
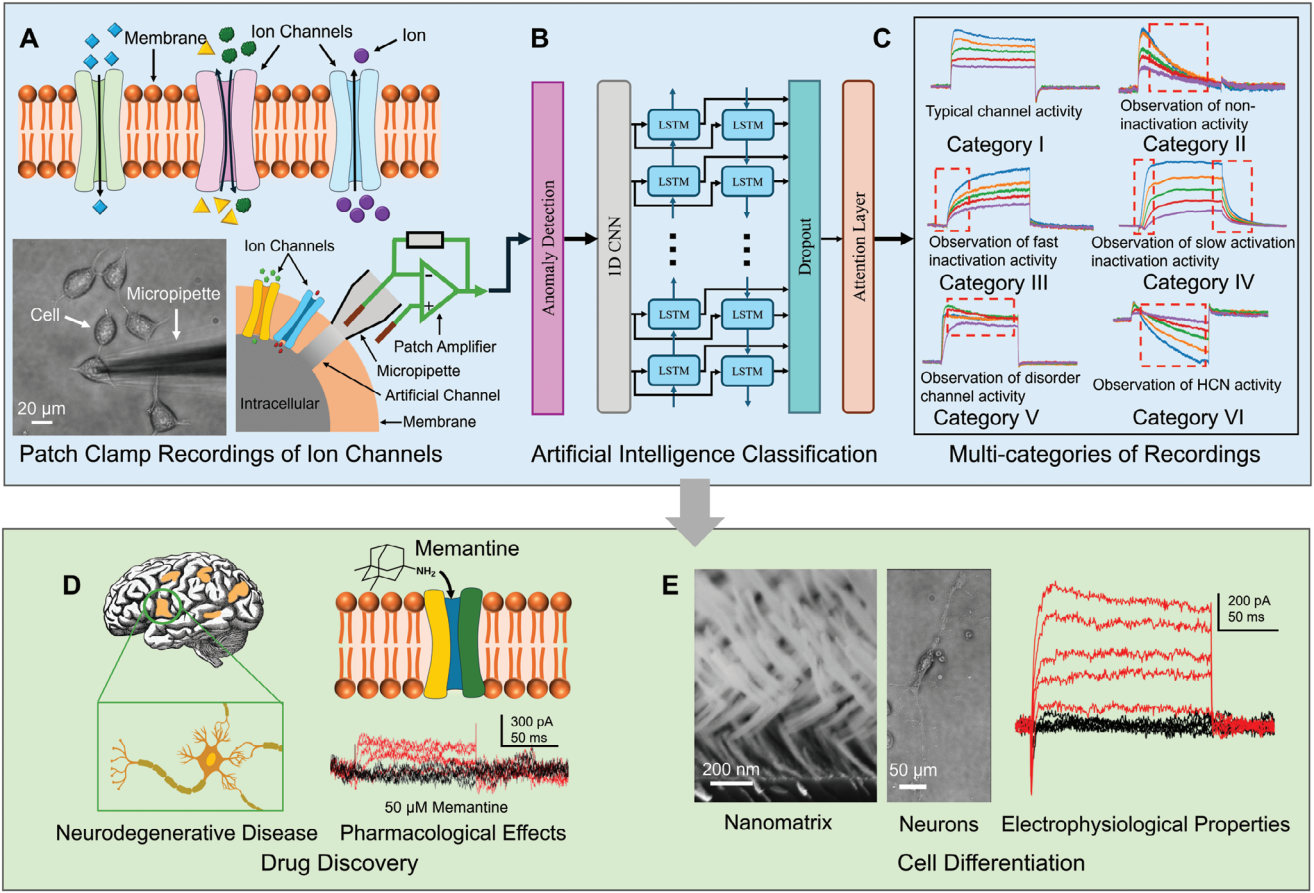
Overview of the proposed method for ion channel kinetics analysis. A) Ion channels on the cell membrane and the whole‐cell configuration for acquiring ion channel currents. B) Anomaly detection for filtering out anomalous recordings and neural networks for recording multi‐class classification. C) Six representative traces of whole‐cell voltage‐clamp recordings: (I) typical ion channel activity; (II) unsustainable outward currents of non‐inactivation activity; (III) slow‐rising current due to the absence of fast inactivation activity; (IV) the presence of slow activation/inactivation activity; (V) disorder channel activity with overlapping currents; and (VI) observation of hyperpolarization‐activated cyclic nucleotide‐gated activity. D) The artificial intelligence framework analyzed recordings to assess the inhibitory effects of memantine on endogenous ion channels, providing kinetics data for drug screening in neurodegenerative diseases. The evoked ion channel activities (red curves) and non‐evoked response currents (black curves) illustrate the effects of the drug. E) The artificial intelligence framework investigated nanomatrix‐induced NSCs differentiation by identifying neurophysiological properties. The activity of evoked sodium and potassium ion channels indicates the functional properties of the neuronal cells.

Artificial intelligence frameworks have been employed in the study of ion channel kinetics. Ion channels span the cell membrane, facilitate ion movement, and regulate various physiological processes (**Figure** **1**a). Protein molecules determine the ions that pass through the ion channel and their kinetics. A micropipette forms a measurement channel to record the ionic current and membrane potential in whole‐cell patch clamp configuration. To analyze ion channel kinetics of whole‐cell recordings, the artificial intelligence framework involves two stages (Figure 1b). In Stage 1, the anomaly detection excludes recordings that are incompatible with typical ion channel behaviors. In Stage 2, the 1DCNN‐BiLSTM‐Attention classifier performs multi‐classification on the recordings that pass anomaly detection to elucidate the kinetics of multiple ion channels. Details of the anomaly detection and the 1DCNN‐BiLSTM‐Attention classifier are explained in Sections 2.2 and 2.3.2, respectively.

Patch clamp recordings have been categorized into specific six categories based on their electrical excitability, kinetic behaviors, and signal features (Figure 1c). The colored lines represent the current traces of ion channels under vary voltage stimuli (Figure S1, Supporting Information).

1. Category I: Representative recordings illustrate the characteristics of ion channels in normal cells.^[^ ^40^ ^]^ The activation behaviors of ion channels vary with different voltage stimuli. High voltage stimuli reveal fast inactivation potassium channels, whereas low voltage stimuli unveil slow inactivation potassium channels. The observed modest inward current indicates low conductance in endogenous sodium and calcium channels. The whole‐cell recording of Category I exhibits typical kinetics for a variety of ion channels.
2. Category II: Non‐inactivation channels are crucial for maintaining the resting membrane potential and regulating cellular excitability.^[^ ^41^ ^]^ For example, when dysfunctional, the absence of sustainable outward (potassium) currents disrupts action potential propagation and impairs muscle contraction. This can prevent signal transmission in neural circuits, potentially leading to cardiomyopathies and epilepsy.
3. Category III: Fast inactivation channels tightly regulate the duration and intensity of cation flow during cellular activities. Failure of fast inactivation prolongs the repolarization phase of the action potential, reducing excitability and signal transmission.^[^ ^42^ ^]^ These impacts can manifest in various tissues, contributing to periodic paralysis and endocrine dysfunction.
4. Category IV: Slow gating kinetics reduce the sensitivity of ion channels, which may result from factors such as mutations in the human ether‐a‐go‐go‐related gene (hERG) potassium channels, as well as in calcium and chloride channels.^[^ ^43^ ^]^ Slow activation delays the cellular response to depolarizing inputs, causing neurons to fire action potentials less frequently, diminishing signal transmission in neural circuits. Moreover, slow inactivation of calcium channels can lead to prolonged calcium entry into presynaptic terminals, potentially causing myotonia and cardiac arrhythmias.
5. Category V: Disordered channel activity leads to overlapping currents, where multiple ionic currents are activated simultaneously or in close succession.^[^ ^44^ ^]^ The relationships among multiple response currents characterize the voltage dependence of ion channel activation and inactivation, as well as ion channel selectivity. This approach uses response currents under different stimulus voltages to evaluate ion channel kinetics and stability and to identify desensitization. In pharmacological testing, repeated voltage steps reveal ion channel behavior over time, providing insights into the consistency of ionic currents.
6. Category VI: In addition to ion channels activated by depolarization, hyperpolarization‐activated cyclic nucleotide‐gated (HCN) channels are activated when the cell membrane potential becomes more negative (hyperpolarizes).^[^ ^45^ ^]^ HCN channels contribute to the control of resting membrane potential and play critical roles in maintaining physiological stability and rhythmicity, such as the heart's rhythmic contractions. Analyzing their kinetics helps in understanding the stabilization of resting potential and cell excitability. Dysfunction in these channels can contribute to various pathophysiological conditions, including seizures and altered pain perception.

Characterizing the functional and electrophysiological properties of cells is a fundamental approach to demonstrate the capabilities of the proposed artificial intelligence framework. We have designed and conducted experiments utilizing our novel artificial intelligence‐assisted ion channel kinetics analysis for two different applications: 1) drug screening for Alzheimer's disease and 2) characterization of nanomatrix‐induced NSCs differentiation. Details of the two applications are provided in Sections 3.1 and 3.2, respectively. For the first application, the exact etiology of Alzheimer's remains unclear. One potential pathology is that excessive glutamate levels may induce excitotoxicity, resulting in neuronal damage. Memantine partially blocks certain receptors to slow the progression of Alzheimer's disease by binding within the receptor's ion channel (Figure 1d). The interaction between memantine and endogenous receptors is a complex research area still under active investigation. Recent studies have identified several potential pathological pathways, particularly involving endogenous channels.^[^ ^26^, ^46^ ^]^ Our deep learning‐based multi‐class classification is a direct and robust method for quantitatively analyzing the effects of memantine on endogenous ion channels. Analyzing the evoked response currents (the red curves) and the absence of ion channel activity (the black curves) allows for the determination of activation thresholds and reversal potentials. This facilitates a deeper understanding of the pathological effects of memantine, providing insights into its therapeutic potential and mechanisms of action. For the second application, the functional differentiated neurons advance the development of cell‐based therapies for neurodegenerative diseases like Parkinson's disease.^[^ ^47^, ^48^ ^]^ We have proposed a nanomatrix that induces the differentiation of NSCs into neurons (Figure 1e). Analysis of whole‐cell recordings was performed to evaluate the effectiveness of the nanomatrix‐induced protocol and the electrophysiological characteristics of neurons. The activities of evoked sodium and potassium ion channels (the red curves) demonstrate that the nanomatrix specifically differentiates NSCs into miniature substantia nigra‐like structures for Parkinson's disease treatment.

### 2.2 Anomaly Detection of Recordings

**Figure 2:**
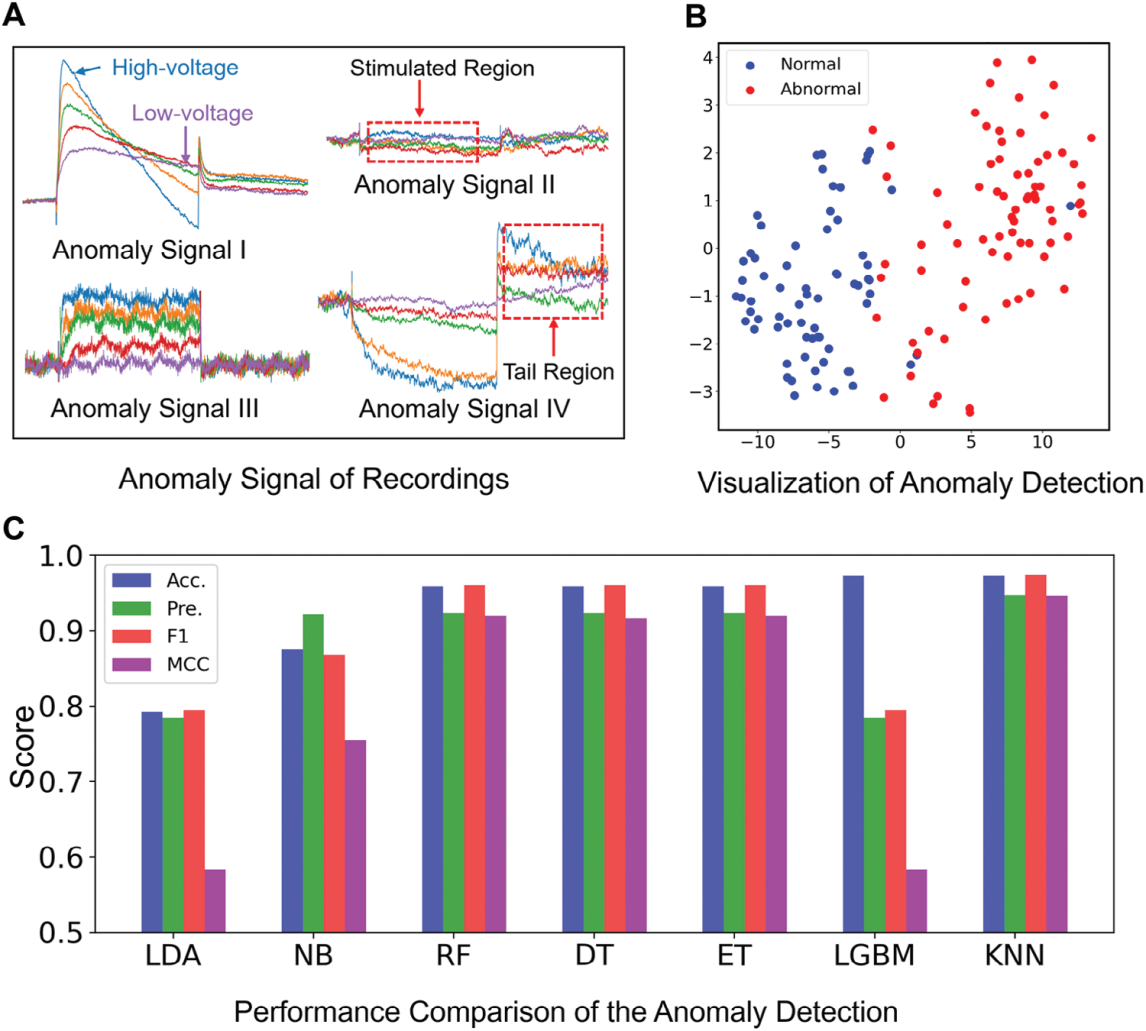
Anomaly detection of recordings. A) Four representative categories of anomaly signal recordings: (I) aberrant transient ion channel behaviors under voltage stimuli; (II) invalid recordings that fail to capture ion channel activity, where the stimulated region shows no discernible correlation between current and voltage; (III) low signal‐to‐noise ratio recording; and (IV) recording with partially abnormal ion channel activity, where erratic depolarization supersedes hyperpolarization in the non‐stimulated tail region. B) Visualization of anomaly detection using principal component analysis (PCA). The coordinates are principal components. The test dataset consists of 72 abnormal and 72 normal recordings. C) The performance comparison of anomaly detection models employed various mathematical approaches: linear discriminant analysis (LDA), naive Bayes (NB), random forest (RF), decision tree (DT), extra trees (ET), light gradient boosting machine (LGBM), and k‐nearest neighbors (KNN). Performance is evaluated using four metrics: accuracy (Acc.), precision (Pre.), F‐score (F1), and Matthew's correlation coefficient (MCC).

Classification accuracy depends on the integrity and authenticity of the recordings representing ion channel dynamics. Before the recording classification, anomaly detection was carried out to remove some outliers, where there were four typical anomaly signals caused by various mechanisms (**Figure** **2**a).

1. Anomaly Signal I: Aberrant transient recording with inconsistent ion channel behaviors is inappropriate for characterizing cellular physiological activities.^[^ ^49^ ^]^ The response current of low‐voltage stimuli revealed the presence of slow/non‐inactivation potassium channels, which were notably absent under high‐voltage stimuli. For the remaining response currents, the response patterns of the inwardly rectifying potassium channels contributing to the outward current exhibited distinct differences among various voltage stimuli. The aberrant transient recordings are insufficient to elucidate the ion channel activity.
2. Anomaly Signal II: Electrical stimulation may evoke disordered ion channel activity, resulting in invalid recordings.^[^ ^50^ ^]^ In the stimulated region, there is no discernible correlation between current and voltage, and the activity of voltage‐gated ion channels cannot be determined from these recordings. This outcome reflects either non‐viable or unhealthy cells, or suboptimal experimental conditions, such as compromised membrane integrity and poor seal quality.
3. Anomaly Signal III: Environmental interference or equipment defects may introduce noise into the recordings.^[^ ^51^ ^]^ With a low signal‐to‐noise ratio, the noise obscures the electrical signals of ion channels, rendering the recording unsuitable for monitoring cellular activity. As a result, the kinetic characteristics of ion channels, including activation threshold, reversal potential, and current density peak, cannot be analyzed.
4. Anomaly Signal IV: Recordings affected by anomalous local ion channel activity have limited utility in characterizing electrophysiological properties.^[^ ^52^ ^]^ Erratic depolarization supersedes hyperpolarization in the non‐stimulated tail region. The resting current associated with this action potential does not match the electrophysiological activity.

A weighted KNN with Euclidean distance was employed to develop an anomaly detection method for identifying recordings that significantly deviate from typical ion channel kinetics. The training dataset included 120 normal recordings distributed over the six categories depicted in Figure 1c (labeled as normal), and an additional 120 abnormal recordings spread across the four categories shown in Figure 2a (labeled as abnormal).

Suppose the training dataset is

(1) $$T={\left\{\left(x_{i},\;y_{i}\right)\right\}}_{i=1}^{N}$$

where *x_i_* ∈ ℜ^*m*^ is the vector in the m‐dimensional feature space and *y_i_* is the normal/abnormal label.

For a query recording *x*′, a set of *k* similar labeled target neighbors were identified. The dataset is

(2) $$T^{\prime }={\left\{\left(x_{i}^{NN},\;y_{i}^{NN}\right)\right\}}_{i=1}^{k}$$

Use the following expression to arrange in an increasing order for distance:

(3) $$d\left(x^{\prime },\;x_{i}^{NN}\right)=\sqrt{{\left(x^{\prime }-x_{i}^{NN}\right)}^{T}\left(x^{\prime }-x_{i}^{NN}\right)}$$

The label of query *y*′ is given by the majority voting:

(4) $$y^{\prime }=\arg\;\max_{y}\;\sum_{\left(x_{i}^{NN},\;y_{i}^{NN}\right)\in \;T^{\prime }}\delta \left(y=y_{i}^{NN}\right)$$

where $y_{i}^{NN}$ is the label for *i*‐th among the KNNs. $\delta (y=y_{i}^{NN})$ is a Dirac delta function that takes the value of 1 or 0.

The test dataset comprised 144 recordings. Using the trained model for anomaly detection yields an accuracy of 97.22%. The detection result underwent dimensionality reduction using principal component analysis (PCA) to extract the main feature components (Figure 2b). The clear separation between the two clusters underscores the efficacy of the anomaly detection method in differentiating between normal and abnormal recordings.

Multiple anomaly detection models were trained and compared to determine the best‐performing implementation. In the model training procedure, various mathematical approaches were utilized to develop the detection algorithms specifically for whole‐cell recording. The trained models are illustrated in Figure 2c. The KNN method outperformed the others across the four key metrics. The KNN‐based method achieved the highest accuracy of 0.9722 and led to an F1 score of 0.9723, demonstrating an optimal balance between precision and recall. The Matthews correlation coefficient (MCC) value (0.9459) further indicated its effectiveness in accurately classifying normal and abnormal recordings. In contrast, while the light gradient boosting machine (LGBM) matched KNN in terms of accuracy, it underperformed in other metrics, particularly with an MCC value of 0.5836. The precision (0.7838) and F1 score (0.7945) highlighted the challenges in detecting anomalous recordings. Extra trees (ETs) comprise multiple decision trees (DTs). Unlike DT and random forest (RF), which utilize the optimal splitting point when a tree splits a node, ET employs a random selection of the splitting point of the feature to mitigate overfitting. Consequently, the ET and similar algorithms have demonstrated notable potential. ET has high performance across several metrics, including accuracy (0.9583) and F1 score (0.9231). Linear discriminant analysis (LDA) and naive Bayes (NB) displayed lower performance, with accuracy and MCC values falling below 0.9 and 0.8, respectively. The metric values indicate difficulties in accurately distinguishing the recordings. The proposed KNN method significantly improved the effectiveness of anomaly detection. Anomaly detection enhances the quality of recordings for further ion channel kinetics analysis by filtering out physiologically irrelevant recordings.

### 2.3 Development of Deep Learning Model

#### 2.3.1 Data Preprocessing Segmentation

**Figure 3:**
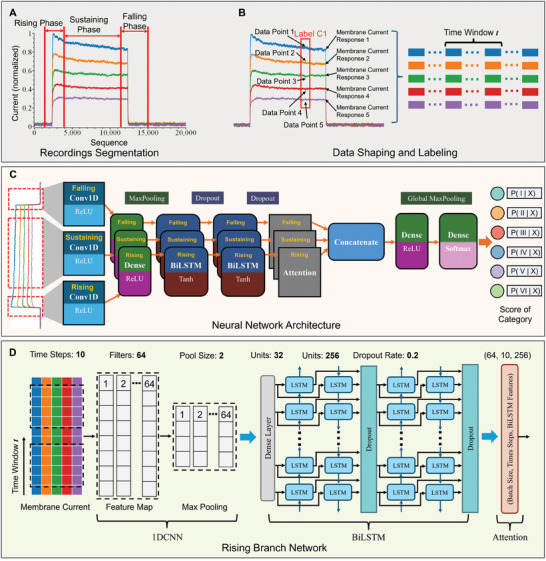
Feature extraction and neural network architecture for multi‐class classification. A) The recording is segmented into three phases: rising, sustaining, and falling. The rising phase marks the increase in current due to voltage‐gated ion channel activation. The sustaining phase presents a steady current, reflecting the equilibrium of ion channel activity. In the falling phase, the end of voltage stimuli manifests as a hyperpolarization process. B) The five response currents are divided into data points within a defined time window, *t*. Each signal sequence captures time‐dependent ion channel activity and current variations resulting from differing voltages. C) Overview of the neural network architecture. Three 1DCNN‐BiLSTM‐Attention branches process each phase independently. The concatenated outputs from the attention layers across all phases yield the whole‐phase recording label. D) Details of rising branch. Time windows segment each phase into multiple time steps. A sliding kernel across these points forms a feature map, subsequently reduced by pooling. After the dense layer, the BiLSTM layers capture temporal dependencies in both directions. Dropout neurons reduce overfitting. An attention layer assigns weights to the recording's time windows, focusing the model on pertinent features.

An ion channel dynamic‐based segmentation strategy was used to extract features from the recordings. Voltage stimuli evoke ion channel activity, leading to distinct stages of action potentials: initial depolarization, transition to repolarization, and final hyperpolarization. After the Z‐score normalization of the raw data, the recording was segmented into three phases according to the dynamics of the action potential: rising, sustaining, and falling (**Figure** **3**a). The rising phase provides insights into the activation of voltage‐gated ion channels, detailing their kinetics and voltage‐dependent behavior. This phase captures both the inward and outward currents. The rapid influx of sodium ions contributes to the upstroke of the action potential, whereas calcium and chloride influxes contribute to cellular processes and membrane potential stabilization. Potassium channels are activated later in this phase to generate an outward current. The sustaining phase characterizes activation/inactivation kinetics and the cellular equilibrium state. During this phase, the delayed rectifier potassium channels are pivotal for maintaining the outward current for the repolarization of the action potential. In addition, calcium‐activated potassium channels are activated in response to increased intracellular calcium levels. Furthermore, ligand‐gated ion channels can sustain currents when continuously exposed to specific ligands. These ion channels significantly contribute to the repolarization of the membrane potential and the regulation of cell excitability. In the falling phase, the deactivation or closure of ion channels highlights their voltage‐dependent deactivation and facilitates the identification of specific ion channels. This phase ends the action potential cycle and reinstates the cell's resting state through transient receptor potentials and hyperpolarization‐activated channels. Deactivation of potassium, sodium, and calcium channels contributes to membrane potential repolarization and hyperpolarization. The ion channel types and kinetics were identified by comparing the inactivation patterns with those observed during the rising and sustaining phases.

In addition to capturing the ion channel characteristics within individual response currents, the model must learn the correlation between multiple response currents. The recordings were segmented into multiple parts within a designated time window (Figure 3b). Because data points within a time window share an identical label, there are two dimensions of variation: 1) time‐dependent ion channel temporal dynamics, and 2) current variations based on voltage‐dependent ion channel activity. Incorporating the response currents evoked by various voltage stimuli within a specific time window allows the model to concentrate on localized patterns and discern interrelations among currents. This approach enables the model to infer the short‐term trends and dynamics of ion channels. Moreover, employing time windows aids in circumventing issues such as vanishing and exploding gradients. The phase boundaries and size of the time window were experimentally determined to optimize the performance of the deep learning model.

#### 2.3.2 Neural Network Architecture

We designed a 1DCNN‐BiLSTM‐Attention deep learning model to capture the complex nonlinear spatial and temporal relationships of whole‐cell voltage‐clamp recordings for multi‐class classification. The neural network architecture is shown in Figure 3c. This architecture employs an independent 1DCNN for each recording phase to extract spatial features and correlations among multiple response currents. These local patterns identify various ion channels and characterize the ion channel dynamics. Subsequently, the features identified by the 1DCNN are processed using a BiLSTM layer to capture the long‐term dependencies of the recordings. This layer amalgamates spatial, temporal, and contextual patterns, elucidating the voltage dependency, kinetics, and variability in response currents. An attention mechanism is implemented to prioritize the features in the BiLSTM output that are most pertinent to the classification label. A dropout rate of 0.2 is implemented to force the model to diversify its reliance across multiple neurons to mitigate overfitting. The concatenation and dense layers integrate features from three independent analyses to label the whole‐phase recording.

The fundamental structure and layers of the neural network are shown in Figure 3d. The 1DCNN framework consists of three primary components: Conv1D, MaxPooling1D, and dense layers. The Conv1D layer employs a sliding kernel to extract spatial features. It generates a feature map by applying a weight kernel matrix to the recordings. The MaxPooling1D layer downsamples the Conv1D output by selecting the maximum value across a specified window of multiple response currents. This process reduces the number of model parameters required to improve training efficiency and enhance noise robustness. Subsequently, the dense layer aggregates the spatial features derived from the Conv1D layer into a more abstract data representation. The weights of the dense layers are refined during training. The BiLSTM network was designed to capture the temporal dependencies in the response currents. Each BiLSTM layer consisted of two distinct LSTM layers, oriented forward and backward. This bidirectional approach enabled the concatenation of outputs from both LSTM layers to capture the time‐series activity of the ion channels. The spatial features identified by the 1DCNN layer were amalgamated with the temporal dependencies characterized by the BiLSTM network. These amalgamated features were fed into a fully connected layer for multi‐class classification. An attention mechanism was deployed to focus on the critical features indicative of ion channel behavior. This mechanism assigns a specific weight to each time window, based on its relative importance. These weights prioritize the most significant features of the BiLSTM output. Subsequently, a global max pooling layer selects the maximal value to encapsulate the spatial features into a cohesive global representation. A fully connected layer then uses this representation to generate a vector of logits. The result indicates the probability that a recording appears in a respective category.

#### 2.3.3 Training and Evaluation

We achieved state‐of‐the‐art results in a multi‐class classification of whole‐cell voltage‐clamp recordings. Performance optimization of the neural network was conducted using a grid search to evaluate all possible hyperparameter combinations. Various training epochs were applied to ensure that the model converged to an optimal solution. The dataset was randomly divided into training and validation sets in an 8:2 ratio for the training process. The model performance was evaluated using a holdout test set.

**Figure 4:**
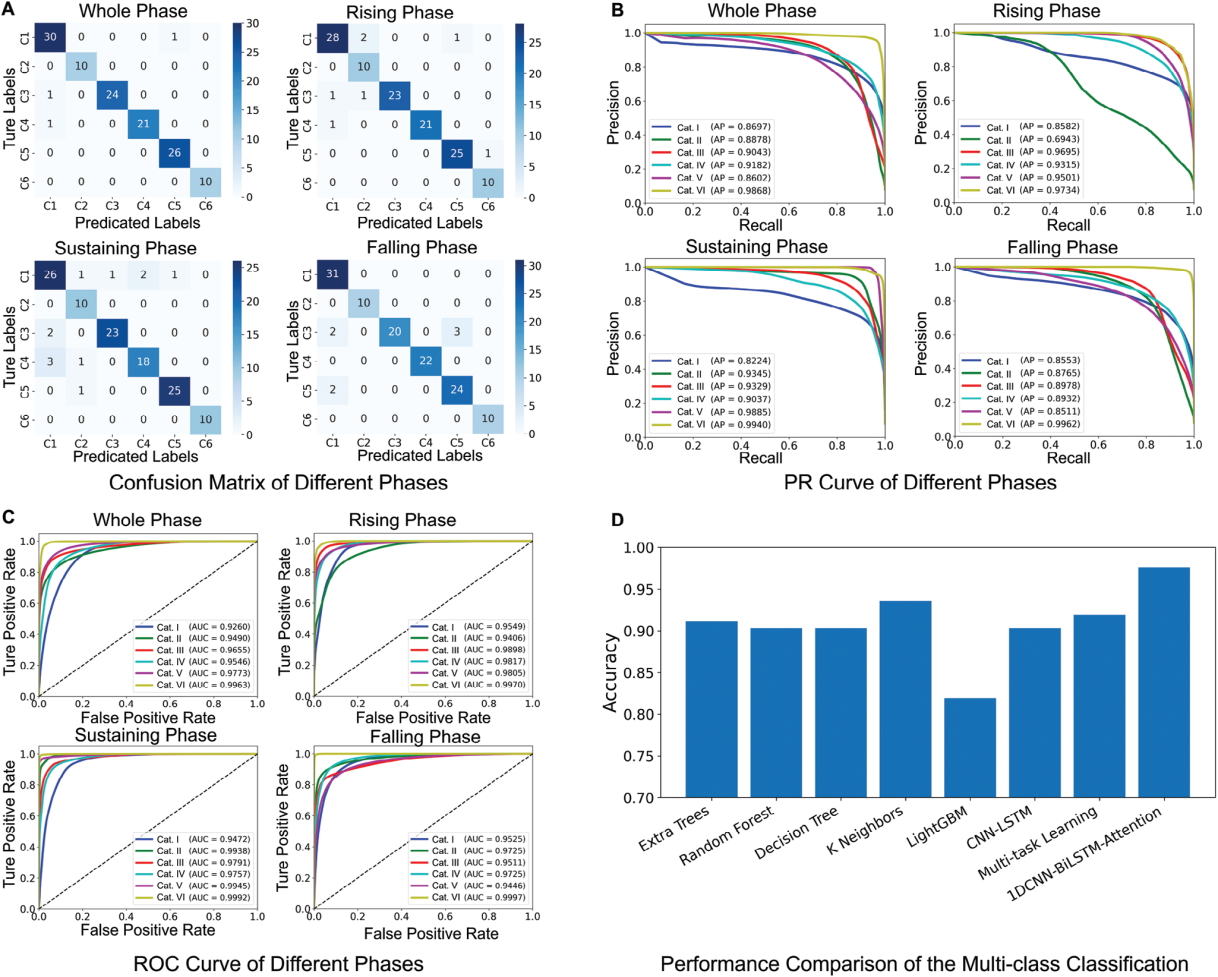
Evaluation and comparison of the 1DCNN‐BiLSTM‐Attention model. The entire phase represents the final output from the deep learning model, while the remaining matrices pertain to segmented phases. The model evaluation metrics include A) confusion matrices, B) precision‐recall (PR) curves, and C) receiver operating characteristic (ROC) curves, along with average precision (AP) and area under the curve (AUC) values. D) Comparison of accuracies obtained from different models using the test set.

The deep learning model demonstrated a prediction accuracy of 97.58% for the test dataset. The confusion matrices are shown in **Figure** **4**a. Different categories may contain similar ion channel behaviors; therefore, the recordings were segmented into multiple phases for classification. The results of the three phases were combined using a deep learning model to reduce the likelihood of misclassification. The prediction accuracies for the three recording phases were 94.35%, 90.32%, and 94.35%, respectively. Categories I, III, and IV exhibited similar ion channel characteristics in the sustaining phase, that is, five parallel response currents. This similarity leads to misclassification of Category I recordings into Categories III and IV in the confusion matrix of the sustaining phase. Consequently, the prediction accuracy for the sustained phase was relatively low. All the prediction results were aggregated using majority voting with an accuracy threshold. By introducing weights after the attention layer to refine the prediction of the entire phase, the final prediction accuracy was 97.58%. These results demonstrate the potential of deep learning for multi‐class classification of whole‐cell voltage‐clamp recordings and illustrate the effectiveness of our model in distinguishing between different recordings.

The average precision (AP) score of 0.9852 indicated the model's performance in terms of both precision and recall. The individual precision‐recall (PR) curve for each category within a phase allows evaluation of the model's performance and the PR tradeoff. The PR curves of the entire phase indicated a consistent balance between precision and recall across varying threshold levels (Figure 4b). Each recording phase yielded independent results with AP scores of 0.9862, 0.9293, and 0.9950 for the rising, sustaining, and falling phases, respectively. The rising phase focused on the behavior of voltage‐gated sodium channels and fast‐inactivation potassium channels. Due to the similarity in ion channel activities, Categories I and II had relatively low AP values, with scores of 0.6943 and 0.8582, respectively. However, the distinction between the channel behaviors in Categories I and II during the sustaining and falling phases improved the model's overall classification accuracy for these categories. The AP scores for the other categories during the rising phase ranged from 0.9315 to 0.9734, demonstrating the model's classification ability. In the sustaining phase, AP scores varied from 0.8224 to 0.9940. The PR curves indicated a high capture rate of true positives and a clear distinction between the absence of slow/non‐active ion behavior. These results determine whether the cells are in a functional physiological state and provide steady electrophysiological responses to stimuli. The PR curves of the falling phase were notably smoother without significant fluctuations, and all AP values exceeded 0.85. This indicates the performance of the model in classifying hyperpolarization‐associated ion channel activities such as those observed in HCN channels. Collectively, the PR curves and AP scores confirmed that the model reliably differentiated between categories.

Receiver operating characteristic (ROC) curve analysis was conducted to evaluate the performance of the model in classifying various ion channel activities across each phase. The area under the curve (AUC) values for the entire phase range from 0.9260 to 0.9963 (Figure 4c). The AUC for the rising, sustaining, and falling phases ranged from 0.9406 to 0.9997. These high AUC values demonstrate the ability of the model to accurately distinguish between true and false positives. Category VI consistently outperformed all phases, with an AUC value greater than 0.99. Although Category I was the lowest‐performing category, it achieved an AUC greater than 0.94. This conclusion is consistent with the insights gained from the PR curve analysis. These findings support the potential utility of this model as a novel method for categorizing whole‐cell recordings.

We benchmarked our deep learning model against various machine learning models (Figure 4d). Notably, due to there is no existing model for multi‐classification ion channel kinetics of whole‐cell recordings, we developed various machine learning algorithms and trained the models specifically for the ion channel kinetics analysis. The optimal parameters for all the comparison models were determined using the grid search method. Constrained by their abilities to capture complex patterns, traditional machine learning models have limitations in the multi‐class classification of intricate recordings. DT‐based models achieved accuracies of approximately 0.9, whereas KNN models fared slightly better, with an accuracy of 0.93. Our deep learning model surpasses these models with an accuracy exceeding 0.97. The performance of the various trained models was systematically evaluated against multiple metrics, as listed in **Table** **1**. The ET classifier performed notably well, with an accuracy of 0.9114, an exceptional AUC of 0.9994, and impressive recall and precision scores of 0.9811 each. These metrics indicated their efficiency in correctly identifying true positives and minimizing false positives. Despite the superior accuracy of KNN, its performance in other metrics suggests the potential for misclassification of ion channel activity. The light‐gradient boosting approach yielded a comparatively lower accuracy of 0.8194 with noticeably inferior performance across the recall, precision, and F1 score metrics. In contrast, our 1DCNN‐BiLSTM‐Attention model excelled across all evaluated metrics, with an accuracy of 0.9758 and an AUC of 0.9994. The F1, Kappa, and MCC indicated the robustness of our model against overfitting and confirmed its superiority in terms of precision and recall. These results highlight the effectiveness of our deep learning model for the multi‐class classification of whole‐cell voltage‐clamp recordings and the potential of the 1DCNN‐BiLSTM‐Attention architecture for real‐world application deployment.

**Table 1:** Comparison of evaluation metrics across multiple models.

Model^a)^	Accuracy	AUC	Recall	Precision	F1	Kappa	MCC
Extra Trees^[^ ^53^ ^]^	0.9114	0.9994	0.9811	0.9811	0.9811	0.9767	0.9767
Random Forest^[^ ^54^ ^]^	0.9032	0.9991	0.9751	0.9750	0.9750	0.9693	0.9693
Decision Tree^[^ ^55^ ^]^	0.9032	0.9686	0.9492	0.9492	0.9492	0.9374	0.9375
Light Gradient Boosting^[^ ^56^ ^]^	0.8194	0.9664	0.8114	0.8117	0.8109	0.7673	0.7674
K‐Nearest Neighbors^[^ ^57^ ^]^	0.9355	0.9646	0.9667	0.9680	0.9667	0.9358	0.9361
CNN‐LSTM^[^ ^58^ ^]^	0.9633	0.9987	0.9633	0.9634	0.9633	0.9480	0.9548
Multi‐Task Learning^[^ ^59^ ^]^	0.9194	0.9998	0.9869	0.9869	0.9869	0.9839	0.9839
1DCNN‐BiLSTM‐Attention	0.9758	0.9994	0.9757	0.9758	0.9757	0.9701	0.9701

^a)^ The citations refer to the original machine learning approaches, there are no existing models for whole‐cell recording analysis.

## 3 Application of Ion Channel Kinetics Analysis

### 3.1 Drug Screening for Alzheimer's Disease

**Figure 5:**
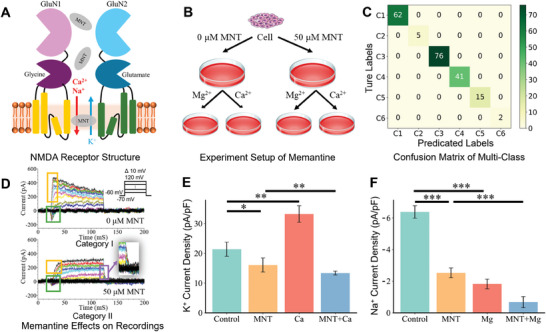
Drug screening of memantine on endogenous ion channels. A) The inhibitory effect of memantine on the NMDA receptor in reducing excitotoxicity associated with Alzheimer's disease. B) The preparation for whole‐cell voltage‐clamp recordings with six different cell culture and extracellular solution sets, including conditions with and without 50 µM memantine and additional sets with 20 mm calcium and 10 mm magnesium. C) The testing results of multi‐class classification by the ion channel kinetics analysis framework. D) Representative whole‐cell voltage‐clamp recordings from cells untreated and treated with 50 µM memantine. The green box indicates the inward sodium current during the rising phase, the outward potassium current during the rising phase by the yellow box, and the tail current during the falling phase by the purple box. E) Potassium current density for the control (n = 28), membrane (MNT, n = 31), calcium (Ca, n = 24), and the memantine and calcium group (MNT+Ca, n = 26). F) Sodium current density for the control (n = 20), memantine (n = 27), magnesium (Mg, n = 22), and the memantine and magnesium group (MNT+Mg, n = 23). (The data are shown as means ± s.d.; *p < 0.05, **p < 0.01, ***p < 0.001.).

The artificial intelligence framework demonstrated its capability for ion channel kinetics analysis in drug screening. Memantine inhibited the overactivation of NMDA receptors by glutamate (**Figure** **5**a), thereby decreasing Na^+^ and Ca^2+^ influx across the cell membrane. However, the effects of memantine on potassium and sodium ion channel activity, as well as the levels of calcium and magnesium ions, have been identified as potential pathological pathways. The undefined dynamics of ion channels limit the therapeutic application in neurodegenerative diseases. HEK 293 cells provide well‐established whole‐cell recordings for the validation of the framework. Additionally, they lack endogenous glutamate receptors but express various cellular signaling components. These components include G proteins, which transmit signals from the extracellular environment to the interior of the cell, and postsynaptic density protein 95 (PSD‐95), a scaffolding protein integral to the clustering of NMDA receptors and potassium channels. The cell samples were divided into two groups: a control group without memantine exposure and a test group treated with 50 µM memantine (Figure 5b). Whole‐cell voltage‐clamp recordings from both groups were compared to evaluate the effect of memantine on endogenous ion channels. Additional groups (both control and test groups) were treated with magnesium and calcium ions to explore the interaction pathways of memantine with endogenous channels.

Recordings from the dominant categories revealed a pronounced inhibitory effect of memantine on endogenous ion channels in HEK 293 cells. The experiment initially collected 292 recordings, of which 240 remained after anomaly detection procedures. These recordings were then classified and analyzed using our framework. Figure 5c presents the final results after excluding 39 recordings with low confidence levels. The recordings were categorized into six categories (Figure 1c), predominantly Categories I, III, and IV, accounting for 179 of the 201 total recordings. A mere 3.48% distribution in Categories II and VI indicates the consistency of ion channel expression in HEK 293 cells.

The recordings revealed characteristic action potentials and electrophysiological behaviors in the presence of memantine. Representative whole‐cell voltage‐clamp recordings from cells treated with and without 50 µM memantine were captured using the ion channel kinetics analysis framework (Figure 5d). In the rising phase of the recordings without memantine, the inward current (primarily from sodium ions) marked the depolarization process. When the voltage stimulus was 60 mV and above, the response currents indicated the presence of fast‐inactivation potassium channels. Slow or non‐inactivation potassium channels contribute to response currents in the sustaining phase. The recordings revealed voltage‐gated potassium ion channel responses to stimuli. The response currents demonstrated that physiological ion transport was facilitated by voltage‐gated ion channels during repolarization. The falling phase showed the inactivation of sodium, potassium, and chloride ion channels. A negligible inward current indicated minimal hyperpolarization. Memantine reduced both inward and outward current amplitudes. Although the duration of the sodium ion current remained unchanged at 15 ms, the negative peak at 60 mV was reduced by 111.36 pA. A significant observation concerning the outward current was the disappearance of the fast‐inactivation potassium current associated with repolarization. This change in the ion channel dynamics reduced the number of outward response currents from nine to seven. The recording exhibited slow gating kinetics in Category IV, indicating inhibited voltage sensitivity of Ca^2+^/Cl^−^ ion channels during hyperpolarization. The pathological pathways may involve interactions between memantine and proteins associated with NMDA receptors. Furthermore, these effects may arise from changes in the phosphorylation status of ion channels.

The effect of memantine on electrophysiological activity was quantified by assessing the relative inhibition of potassium and sodium currents. Memantine led to a 5.29pA pF^−1^ reduction in the outward potassium current density (Figure 5e). In contrast, experiments using a high‐calcium solution showed a notable increase in current density by 11.86pA pF^−1^. Elevated extracellular calcium levels can activate calcium‐activated potassium channels, particularly the large‐conductance, calcium‐activated potassium (BK) channel. Upon the binding of intracellular Ca^2+^ to the RCK1 and RCK2 sites, the BK channel promoted potassium ions across the cell membrane and facilitated repolarization. However, the presence of both memantine and the high‐calcium solution intensified the inhibitory effect on the outward potassium ion channels, diminishing the current density to 13.35pA pF^−1^. Memantine reduced cytosolic calcium levels to attenuate BK channel activation. The blocked slow activation and non‐activation of potassium channels suggest an antagonistic interaction between memantine and elevated extracellular calcium that affects potassium channels. This is possibly mediated by downstream signaling pathways or calcium‐dependent mechanisms. Further experimental investigation would be warranted to understand the potential competitive interactions for developing memantine therapeutic strategies. When memantine is synergized with magnesium ions, its efficacy in inhibiting sodium ion channels is enhanced (Figure 5f). Memantine reduced the sodium ion current density by 3.85pA pF^−1^. Although Mg^2+^ ions also serve as pore blockers for sodium channels, their dwell time within the channel is shorter than that of memantine. Applying a high‐Mg solution resulted in a pronounced decrease in the inward current to 1.83pA pF^−1^. The effect of Mg^2+^ on sodium ion channels is use‐ and voltage‐dependent. The inhibitory effect intensified at higher channel activation frequencies and more depolarized membrane potentials. When memantine was combined with a high‐magnesium solution, the affinity of Mg^2+^ for the sodium channel increased significantly, resulting in a marked reduction in the sodium current. Consequently, the current density decreased further to 0.68pA pF^−1^. This synergistic effect resulted in depolarization.

**Figure 6:**
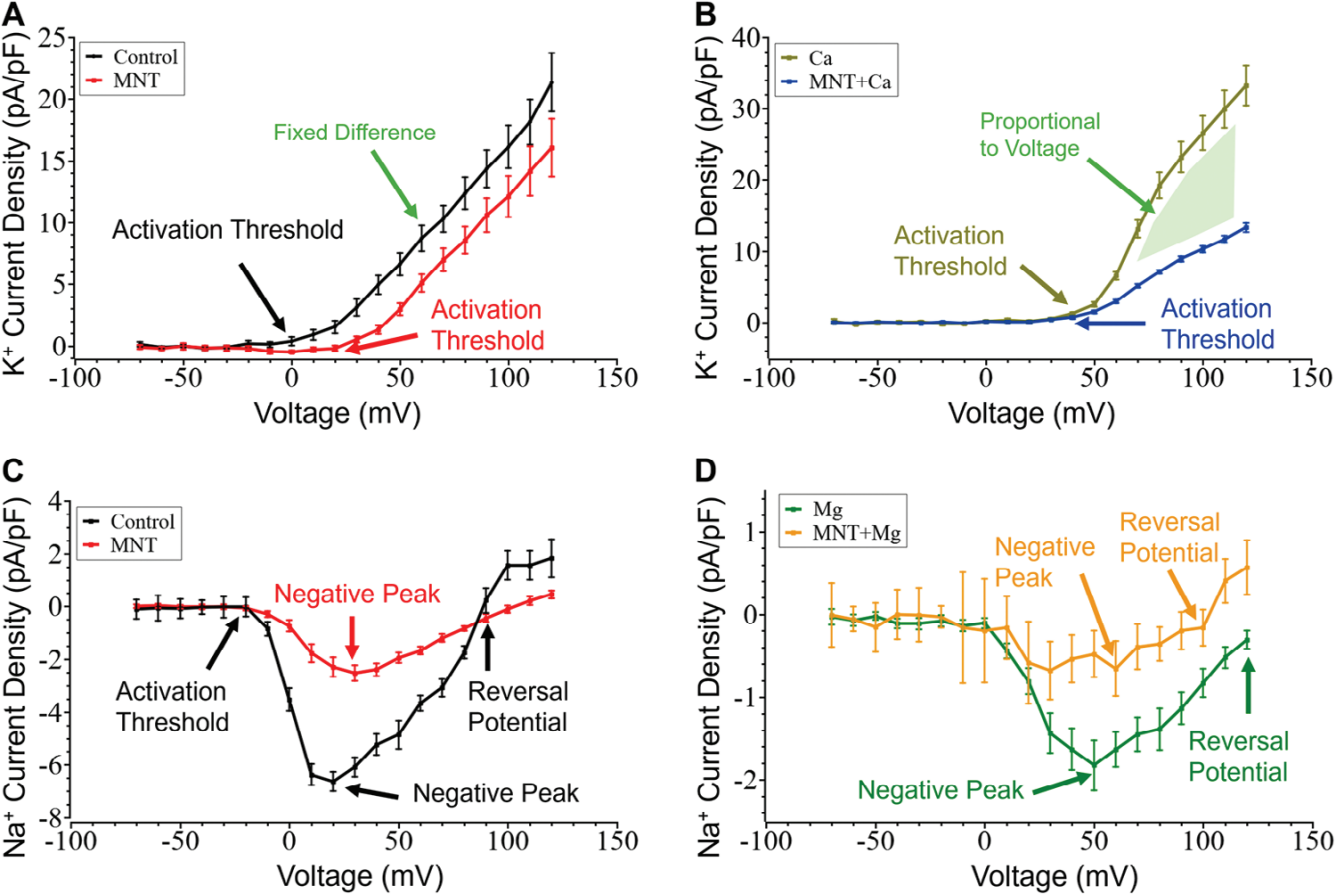
Dynamics of ion channels during memantine (abbreviated MNT in the figure) drug screening. Memantine represents the inclusion of 50 µM memantine in the culture medium. The extracellular solutions contained 10 mm Mg ions or 20 mm Ca ions. The depicted outward current‐voltage (I–V) curves reflect the behavior of potassium (K^+^) ion channels under different conditions: A) MNT (n = 31) versus control (n = 28), and B) Ca (n = 24) versus MNT+Ca (n = 26). Sodium ion channel activities are represented by the inward sodium (Na^+^) *I–V* curves for C) MNT (n = 27) versus control (n = 20), and D) Mg (n = 22) versus MNT+Mg (n = 23). (The data are shown as means ± s.d.).

Based on the ion channel kinetics analysis results, current–voltage (*I–V*) curves were plotted to illustrate ion channel dynamics and electrophysiological properties. The relationship between membrane potential and current through endogenous ion channels is shown in **Figure** **6**. Memantine shifted the activation threshold, as evidenced by an increase in the potential threshold from 0 to 20 mV (Figure 6a). The action potential is inhibited by a more depolarized state for the ion channels to open. A 22% reduction (at 50 mV) in current density further indicated changes in conductance and gating kinetics, suggesting voltage‐dependent inhibition of potassium channels. The high‐calcium solution profoundly affected the activation threshold of the endogenous ion channels (Figure 6b). Obvious potassium channel currents manifested only above 40 mV and show voltage‐dependent conductance. Under high‐calcium conditions, high ion channel conductance indicates a substantial number of calcium‐activated ion channels in HEK 293 cells, which are sensitive to memantine. The *I–V* curve from the memantine and high‐calcium solution groups revealed an antagonistic interaction, suggesting downstream signaling pathways and calcium‐dependent mechanisms for endogenous ion channels.

Memantine significantly inhibited the formation of sodium ion channels during depolarization (Figure 6c). At 0 mV, the current density in the control condition was markedly higher than that in the memantine‐treated condition. Additionally, the negative peak was substantially reduced from −6.64 to −2.52 pA pF^−1^ in the presence of memantine. The observed changes in inward (sodium) and outward (potassium) current densities demonstrated an inhibitory effect on action potential depolarization. In the magnesium‐alone condition, the *I–V* curve conformed to the typical sodium ion channel behavior, with inward currents at negative potentials and outward currents at positive potentials (Figure 6d). The negative peak was reduced to −1.83 pA pF^−1^. The combined memantine and magnesium condition resulted in a pronounced reduction in the inward current at all potentials. The negative peak was further reduced to −0.67 pA pF^−1^. Compared to the conditions shown in Figure 6b, the presence of magnesium necessitates a higher reversal potential. Ion channels become more permeable to ions with positive equilibrium potentials such as sodium and calcium ions. This implied substantial inhibition of sodium channel activation and a consequential absence of action potentials. The *I–V* curves provide insights into the voltage‐gated characteristics of ion channels and their activation and inactivation dynamics, suggesting that memantine can modulate voltage‐dependent inactivation/activation and alter channel conductance.

### 3.2 Nanomatrix‐Induced Cell Differentiation

**Figure 7:**
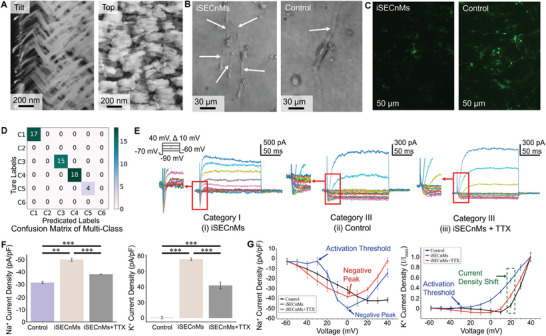
Characterization of the specific phenotypic differentiation of NSCs by iSECnMs. A) SEM images of iSECnMs showing tilt and top views. The zigzag pitch is approximately 170 nm, and the stiffness is 2.04 ± 0.22 GPa (Young's modulus). B) NSCs were seeded on iSECnMs (left) and glass plates (right, control group) to induce differentiation. NSCs morphology was monitored on the 14th day of culture via microscopy. Axon‐like structures were marked by white arrows. C) The differentiation of NSCs mediated by iSECnMs and control was determined using immunocytochemical analysis of GFAP expression (stained in green). The fluorescence intensity was 12.85 A.U. for iSECnMs and 52.79 A.U. for the control. D) The classification results of ion channel kinetics from recordings of differentiated NSCs, as analyzed by the artificial intelligence framework. E) Representative whole‐cell voltage‐clamp recordings from differentiated NSCs on (i) iSECnMs, (ii) the control, and (iii) iSECnMs with TTX. F) Current density of differentiated NSCs on iSECnMs and the control: outward K^+^ current density (left); and inward Na^+^ current density (right). G) *I–V* curves of inward current for the control (n = 12), iSECnMs (n = 15), and TTX (n = 12). Normalized *I–V* curves of outward current for the control (n = 12), iSECnMs (n = 15), and TTX (n = 12). Data are presented as means ± s.d.; *p < 0.05, **p < 0.01, ***p < 0.001.

The artificial intelligence framework demonstrated its capability for effectively characterizing neurophysiological functions and electrophysiological properties. The framework provides a novel, efficient, and automatic method for ion channel kinetics analysis in electrophysiological research, validated using the newly unseen whole‐cell recordings from nanomatrix‐induced differentiation application. Parkinson's disease is characterized by the degeneration of dopaminergic neurons in the substantia nigra (SN) neurons. We engineered inorganic sculptured extracellular nanomatrices (iSECnMs) to specifically differentiate NSCs into miniature SN‐like structures (mini‐SNLSs) intended for cell therapies targeting Parkinson's disease.^[^ ^39^ ^]^ By applying the framework to the iSECnMs‐induced differentiation study, whole‐cell recordings were efficiently and accurately classified for the analysis of ion channel kinetics. The iSECnMs were sculptured into three‐pitch nanozigzags by glancing angle deposition (**Figure** **7**a). The induction of cell differentiation by iSECnMs is attributed to its surface roughness or contact depth.^[^ ^60^, ^61^, ^62^ ^]^ NSCs differentiated on iSECnMs exhibited morphological traits of mini‐SNLSs (Figure 7b). Those cells exhibited more axon‐like structures than the cells on glass plates and were also thicker. These morphological differences indicate a more advanced functional maturation of iSECnMs‐induced differentiation. The iSECnMs enhanced focal adhesion and promoted increased physical contact between the growing cells and the nanostructures. The differentiation of iSECnMs results in neuronal somas that firmly adhere to the substrate and the formation of synaptic connections by axons and dendrites. In contrast, the cells that differentiated on glass plates did not exhibit neuronal characteristics. The NSCs have the potential to differentiate into astrocytes. Astrocytes are characterized by high levels of glial fibrillary acidic protein (GFAP), whereas mini‐SNLSs exhibit minimal GFAP. Immunocytochemical analysis was performed to evaluate NSC differentiation (Figure 7c). iSECnMs tended to reduce the expression of GFAP compared to that in the control group. These results indicate that iSECnMs direct cell differentiation toward mini‐SNLSs instead of astrocytes. Patch clamp experiments were conducted on the two groups of differentiated cells, which yielded 88 recordings. Anomaly detection reduced the dataset to 54. Employing the framework for ion channel kinetics analysis resulted in 39 recordings with high confidence (Figure 7d). The classified recordings primarily fell into Categories I, III, and IV, and all four recordings in Category V belonged to the control group. Category V recordings revealed inconsistent potassium ion channel activity during the sustained phase, suggesting that the glass plate caused limited differentiation. The absence of Categories II and VI demonstrated the capabilities of iSECnMs‐induced differentiated neurons for action potentials. The distribution of recordings across the categories indicated that normal physiological activities were maintained during differentiation.

Whole‐cell voltage recordings conducted on iSECnMs‐induced neurons demonstrated clear inward sodium currents, indicating depolarization, and outward potassium currents, indicating repolarization (Figure 7e). Voltage stimuli between 0 and 40 mV produced five potassium response currents. The control group exhibited a modest inward sodium current and potassium channel activation at 30–40 mV. These ion channel dynamics are consistent with the conclusions regarding GFAP. To assess the specificity of the sodium channels in iSECnMs‐induced neurons, 50 nM tetrodotoxin (TTX) was added to the extracellular solution. TTX selectively blocks voltage‐gated sodium channels, leading to the absence of an inward current. Consequently, the subsequent repolarization was not fully initiated and was triggered only at potentials ranging from 20 to 30 mV. Neurons differentiated on the iSECnMs demonstrated functional properties, particularly action potential generation and nerve impulse conduction (Figure 7f). The sodium ion channel contributes to the depolarization of the action potential. The current density was −50.47 pA pF^−1^, which is higher than the −31.85 pA pF^−1^ observed in the control group. TTX suppresses cell excitability and action potential generation by blocking rapid sodium influx. The −38.56 pA pF^−1^ caused by TTX indicated that the neurons contained voltage‐gated sodium channels involved in action potential initiation and propagation. Voltage‐gated potassium channels are critical for maintaining action potentials. The neurons exhibited K‐current densities as high as 75.94 pA pF^−1^. The control group showed an undetectable potassium current. The blockade of sodium ion channels by TTX impeded the complete opening of potassium ion channels, yielding a reduced current density of only 42.20 pA pF^−1^. The *I–V* curves indicate the ion channel dynamics for neuronal functionality (Figure 7g). The action threshold for neurons differentiated on iSECnMs is −30 mV, with the negative peak observed at 0 mV. As the voltage was increased further, the sodium ion channels gradually underwent inactivation. In comparison, the control group exhibited sodium ion channel activity, which is uncharacteristic of action potentials. The behavior of potassium ion channels reinforced this finding. In the control group, the potassium ion channels were activated only at voltages exceeding 20 mV. The notable shift of 0.51 observed at 20 mV indicated a deviation from the standard neuronal potassium ion channel dynamics. The observed shifts of 0.51 to iSECnMs‐induced neurons and 0.26 to the TTX condition at 20 mV indicate deviations from typical neuronal potassium ion channel dynamics.

## 4 Discussion

Our artificial intelligence framework has effectively advanced the ion channel kinetics analysis of whole‐cell recordings by combining anomaly detection with a deep learning classifier. Previous approaches have predominantly focused on the evaluation of individual ion channels while our approach captures the collective behavior and interactions of multiple ion channels.^[^ ^18^, ^63^ ^]^ The identification of spatiotemporal patterns and features in whole‐cell recordings provides a comprehensive and accurate assessment of cellular electrical properties for drug screening, pathophysiological studies, and neuronal functional characterization. For model development, HEK 293 cells were employed due to their status as a well‐established and widely utilized model system in electrophysiological research.^[^ ^64^, ^65^, ^66^, ^67^ ^]^ This cell line provides a consistent and reproducible platform for the study of ion channel physiology, pharmacology, and the cellular mechanisms underlying electrophysiological processes.^[^ ^68^, ^69^, ^70^, ^71^, ^72^ ^]^ HEK 293 cells express a diverse array of functionally significant endogenous ion channels, such as voltage‐gated potassium channels, voltage‐independent chloride channels, transient receptor potential channels, and store‐operated channels.^[^ ^73^, ^74^ ^]^ The comprehensive kinetic data obtained from whole‐cell recordings of HEK 293 cells ensures the generalizability of the trained model, as demonstrated in the four practical applications. In the validation stage of model training, the validation accuracy increases rapidly during the initial epochs and gradually plateaus at 98.98% (Figure S5, Supporting Information). This learning curve suggests that the model is performing well without significant overfitting or underfitting. The testing accuracy of 97.58% indicates the model's generalization performance on new, previously unseen recordings. In practical use cases, a comparison of the model‐generated confusion matrix with expert‐annotated results shows that the deep learning model achieves an accuracy of 97.01% for ion channel kinetics analysis in drug screening and 92.31% for nanomatrix‐induced differentiation. The artificial intelligence framework innovative integrates anomaly detection and deep learning classifier. KNN‐based anomaly detection enhances data quality by accurately identifying and filtering out anomalous recordings. 1DCNN‐BiLSTM‐Attention classifier addresses ion channel kinetics of whole‐cell recordings by combining multiple architectures. The 1D convolutional layers effectively extract spatial features from the recordings, and capture ion channel activation and inactivation. The BiLSTM layers model the long‐range temporal dependencies present in the recordings. The attention mechanism allows the model to focus on the most relevant input features when making predictions. The proposed 1DCNN‐BiLSTM‐Attention model shows state‐of‐the‐art performance compared with the previous algorithms and models.^[^ ^56^, ^57^, ^58^, ^59^, ^60^, ^61^, ^62^ ^]^ The novel design of the framework and the incorporation of diverse kinetics for model training could meet the current electrophysiological demand for the development of effective and accurate patch clamp recording analysis.

This study has demonstrated for the first time that artificial intelligence enables ion channel kinetics analysis of whole‐cell recordings for practical applications. Four distinct applications underscore the versatility and real‐world impact of the artificial intelligence framework. In Alzheimer's disease drug screening, the framework reveals intricate interactions between memantine and endogenous ion channels. This application suggested potential pathological pathways for drug development. The iSECnMs have been utilized to facilitate neuronal differentiation in various neuroscience studies.^[^ ^75^, ^76^, ^77^, ^78^ ^]^ The iSECnMs are hypothesized to collectively influence cell behavior through their geometrical profile, contact depth, and surface roughness.^[^ ^60^, ^61^, ^62^ ^]^ Due to structural constraints, contact depth is employed as a surrogate metric for evaluation. In the nanomatrix‐induced NSCs differentiation experiments, the framework enhances the efficiency and accuracy of characterizing neurophysiological functions and electrophysiological properties, providing a novel method for signal processing in electrophysiology research. Additionally, we conducted two neuronal experiments utilizing the framework to classify distinct ion channel kinetics (Section S2, Supporting Information). The whole‐cell recordings were sourced from commonly used public datasets obtained from other research groups. The artificial intelligence framework accurately classified the A‐type potassium activity of γ‐aminobutyric acid (GABA) interneurons with 100% accuracy and achieved 93.33% accuracy in identifying fast inactivation potassium channel activity in serotonin (5‐HT) neurons. These new results further verify the model's generalization and success across different recording signals. The demonstrations adhere strictly to standard deep learning producers by employing three types of data: 1) well‐established whole‐cell recordings, 2) public datasets of whole‐cell recordings, and 3) newly unseen whole‐cell recordings.^[^ ^79^, ^80^, ^81^ ^]^ The well‐established whole‐cell recordings facilitate robust classification through diverse and high‐quality data, and enable the development of state‐of‐the‐art models with proven performance. Well‐established whole‐cell recordings were obtained from drug screening for Alzheimer's disease. The kinetic data validated the accuracy and reliability of our artificial intelligence framework in the analysis of multiple ion channel kinetics. Public datasets in deep learning development promote reproducibility, ensure consistency, and facilitate comparability. The public datasets results verified the model's ability to identify different recording signals and provided a consistent standard for evaluating performance. Newly unseen whole‐cell recordings were utilized to assess the model's performance, generalization capabilities, and proficiency in managing real‐world scenarios. The framework characterized neurophysiological functions and electrophysiological properties in the nanomatrix‐induced differentiation, indicating its effectiveness and generalizability in predicting real‐world applications. To achieve the generalizability of the trained model, we designed four whole‐cell voltage‐clamp protocols to evoke diverse ion channel activities, resulting in six representative categories of whole‐cell recordings (Figure S1, Supporting Information). Variations in cellular physiological states and experimental conditions can cause identical protocols to produce different response currents. For instance, in drug screening, two protocols generated recordings across all six categories, whereas another two protocols for matrix‐induced differentiation resulted in recordings within four categories (Figure S2, Supporting Information). These practical applications clearly demonstrate the great potential of our artificial intelligence framework for the achievement of efficient and accurate kinetics analysis in subjects with electrophysiology.

Although our results are promising, there are some limitations. The performance of the artificial intelligence framework depends on the quality and diversity of the training data. Expanding the dataset to include a broader range of cell types and conditions may further improve its accuracy and generalizability. The patch clamp technique has multiple configurations and methods for various studies, such as current‐clamp recording and patch‐sequencing.^[^ ^82^, ^83^ ^]^ Future development will also investigate the applicability of this framework to comprehensive applications. Additionally, exploring the integration of other deep learning architectures and techniques may yield even more robust frameworks.

## 5 Conclusion

In this study, we present a novel artificial intelligence framework for the ion channel kinetics analysis of whole‐cell patch clamp recordings. The framework consists of anomaly detection and a deep learning model. KNN‐based anomaly detection effectively excludes recordings that deviate from the expected ion channel kinetics with an accuracy of 97.22%. Anomaly detection ensures that the deep learning model is trained using a reliable dataset. The 1DCNN‐BiLSTM‐Attention deep learning model accurately identifies ion channel behaviors and cellular electrophysiological properties. We demonstrate a classification accuracy of 97.58% across 124 recordings spanning six categories. The efficacy of the framework is demonstrated through its application in drug screening for Alzheimer's disease and characterization of matrix‐induced stem cell differentiation. In the drug screening study, the framework revealed the inhibitory effects of memantine on endogenous ion channels in HEK 293 cells. These findings provide insights into potential therapeutic pathways and calcium‐dependent interactions. Additionally, the framework validates the functional properties of dopaminergic neurons differentiated from NSCs on nanostructured matrices. The utility of characterizing cellular differentiation protocols is also discussed. The artificial intelligence framework offers a precise, automated, and efficient method for classifying cellular ion channel behavior, thereby addressing the critical need for rapid and accurate data analysis in electrophysiological research. Its application has promising implications for accelerating progress in drug discovery, disease modeling, and the development of therapeutic strategies.

## 6 Experimental Section

### Patch Clamp Recording

Whole‐cell voltage‐clamp recordings were collected using an automated patch clamp system.^[^ ^84^ ^]^

For the development of the artificial intelligence framework, HEK 293 cells were employed to obtain recordings as a dataset for training and validating the artificial intelligence framework. The dataset for anomaly detection training contained 240 recordings, and the testing dataset included 144 recordings. For multi‐class classification, 139 recordings spanning six categories were used for training, and the testing set comprised 124 recordings. The protocols were shown in Section S1, Supporting Information. For the multi‐class classification, the whole‐cell recordings in Categories I, II, and V were obtained using Protocol I. The distinct ion channel activities arise from different cellular physiological variations and states. Protocols II, II, and IV were used to obtain whole‐cell recordings for Categories III, IV, and VI, respectively. For anomaly recordings, Protocol I was used to capture typical anomalous ion channel activity in Anomaly signals I, II, and III. Anomaly Signal IV was obtained by Protocol IV to investigate the inconsistent ion channel activity. The intracellular solution contained (in mm): 75 KCl, 10 NaCl, 70 KF·2H_2_O, 2 MgCl_2_, 10 EGTA, and 10 HEPES, and the pH value was adjusted to 7.2 with KOH. The extracellular solution compounds were (in mm): 160 NaCl, 4.5 KCl, 1 MgCl_2_, 2 CaCl_2_, 5 glucose, and 10 HEPES. The pH value was adjusted to 7.4 with NaOH.

For the drug screening study, a total of 292 recordings were obtained using two voltage‐clamp protocols to investigate the effects of memantine, magnesium, and calcium on sodium and potassium ion channel activities. Protocols VII and VIII were utilized to assess potassium and sodium ion channel activities, respectively. Patch pipettes were filled with intracellular solution (in mm): 6 KCl, 4.6 MgCl_2_, 1.1 CaCl_2_, 113 K‐gluconate, 4 Na_2_‐ATP, 0.4 Na_2_‐GTP, 10 EGTA, and 10 HEPES, and the pH value was adjusted to 7.3 with KOH. The extracellular solution consisted of (in mm): 138 NaCl, 5.3 KCl, 0.5 MgCl_2_, 0.4 KH_2_PO_4_, 0.3 Na_2_HPO_4_, 4.2 NaHCO_3_, 0.4 MgSO_4_, and 2 CaCl_2_. The pH value was adjusted to 7.4 with NaOH. Memantine was purchased from Anaqua, Hong Kong (98% purity). Memantine was dissolved to a concentration of 100 mm and stored at −20 °C. During experiments, the memantine solution was dissolved in an extracellular solution and tested at concentrations of 50 µM. Magnesium chloride and calcium chloride were used to alter the Mg^2+^ and Ca^2+^ concentrations in the extracellular solution.

For the nanomatrix‐induced differentiation of NSCs, 88 recordings were obtained from both the iSECnM group and the control group. Two voltage‐clamp protocols were employed to identify neuronal functional properties and classify electrophysiological characteristics. The voltage‐clamp protocols are shown in Figure S2, Supporting Information. The intracellular solution included (in mm): 107 KCl, 1.2 MgCl_2_, 1 CaCl_2_, 10 EGTA, 5 HEPES, and 3 Mg‐ATP, and the pH value was adjusted to 7.3 with KOH. The extracellular solution consisted of (in mm): 150 NaCl, 5 KCl, 1 MgCl_2_, 2 m CaCl_2_, 10 glucose, and 10 HEPES. The pH value was adjusted to 7.4 with NaOH.

### HEK 293 Cell Culture for Framework Development

HEK 293 (ATCC, CRL‐1573) cells for the ion channel kinetics analysis framework development were maintained in Dulbecco's Modified Eagle Medium (DMEM)/F12 with GlutaMAX (Thermo Fisher Scientific, 10565018) supplemented with 10% fetal bovine serum (FBS; Thermo Fisher Scientific, A3160801), 1 × Penicillin‐Streptomycin‐Neomycin (PSN) Antibiotic Mixture (Thermo Fisher Scientific, 15640055) at 37 °C in a 5% CO_2_ incubator.

### Cell Culture for Drug Screening

HEK 293 (ATCC, CRL‐1573) cells for the drug screening study were maintained in DMEM (Thermo Fisher Scientific, 12430054) supplemented with 10% FBS and 1 × PSN Antibiotic Mixture at 37 °C in a 5% CO_2_ incubator.

### NSC Isolation and Cell Differentiation

The NSCs were isolated from Sprague–Dawley rats (Chinese University of Hong Kong) and cultured in neurobasal medium (ThermoFisher Scientific, 21103049) supplemented with 10% FBS, 2% B‐27 supplement (Thermo Fisher Scientific, 17504044) and 1 × PSN Antibiotic Mixture at 37 °C in a 5% CO_2_ incubator. For differentiation, the cells were cultured on silica nanomatrices using a neurobasal medium supplemented with 10% FBS and 1 × PSN Antibiotic Mixture at 37 °C in a 5% CO_2_ incubator. The silica nanomatrices consisted of SiO_2_‐NZ245‐N_3_. The neural stem cells were seeded on the nanomatrix as the experimental group and glass plates as the control group.

### Generalizability of the Deep Learning Model to Other Neurons

The deep learning model demonstrated its generalizability in the ion channel kinetics analysis of GABA interneurons and 5‐HT neurons (Section S2, Supporting Information). The whole‐cell recordings come from commonly used public datasets obtained from other research groups.^[^ ^85^ ^]^ GABA interneurons are widely distributed throughout the brain and spinal cord, regulating neuronal excitability and maintaining the balance between excitation and inhibition within neural circuits.^[^ ^86^, ^87^ ^]^ Axons of 5‐HT (serotonin) neurons project to various regions of the brain and spinal cord, significantly influencing mood regulation.^[^ ^88^, ^89^ ^]^ The deep learning model analyzed the response currents from A‐type voltage‐gated potassium channels in GABA interneurons and successfully classified the recordings into Categories I, II, and III with 100% (13/13) accuracy. The deep learning model investigated the fast inactivation potassium and non‐inactivation channels involved in the signaling transmission of 5‐HT neurons. It identified dysfunctional ion channel activity with 93.33% (14/15) accuracy, specifically noting the absence and partial activation delays of fast inactivation potassium channels.

### Immunofluorescent Staining of Differentiated NSCs

The cells were fixed with 4% paraformaldehyde (Sigma, PFA) for 30 min at 25 °C. Next, specific dilutions of the primary antibody were incubated overnight at 4 °C in phosphate‐buffered saline containing 0.1% Triton X‐100 (Sigma) and 2% normal goat serum (Vector Laboratories). Subsequently, the cells were stained with specific secondary antibodies for 3 h at 25 °C. After staining, the cells were mounted with a fluorescence mounting medium (Dako) and immunoreactivity was visualized using a confocal microscope (FluoView FV1000, Olympus). The primary antibody used in this study was anti‐GFAP (1:500, AB5804, Millipore).

### Statistical Analysis

Results were presented as mean ± standard deviations (s.d.). Student's t‐test was used to test the differences between two groups affected by a single variable. ANOVA with multiple comparison tests were used to compare multiple groups. All data were analyzed by Python using NumPy and pandas. Statistical significance was defined as *p < 0.05, **p < 0.01, ***p < 0.001, ****p < 0.0001. Details of the n number can be found in the appropriate figure legend. ImageJ (National Institutes of Health, USA) was used for immunocytochemical analysis and cell counting analyses. Detailed statistical values can be found in the figure legends.

## Conflict of Interest

The authors declare no conflict of interest.

## Supporting information

Supporting Information

## Data Availability

The data that support the findings of this study are available from the corresponding author upon reasonable request.

## References

[1] T. Parpaite , L. Brosse , N. Séjourné , A. Laur , Y. Mechioukhi , P. Delmas , B. Coste , Cell Rep. 2021, 37. 10.1016/j.celrep.2021.109914 PMC8578708 34731626

[2] D. Vandael , Y. Okamoto , C. Borges‐Merjane , V. Vargas‐Barroso , B. A. Suter , P. Jonas , Nat. Protoc. 2021, 16, 2947. 33990799 10.1038/s41596-021-00526-0

[3] T. Minakuchi , E. M. Guthman , P. Acharya , J. Hinson , W. Fleming , I. B. Witten , S. N. Oline , A. L. Falkner , Nat. Neurosci. 2024, 24, 702. 10.1038/s41593-023-01563-6 PMC12716102 38347201

[4] C. D. Sessler , Y. Zhou , W. Wang , N. D. Hartley , Z. Fu , D. Graykowski , M. Sheng , X. Wang , J. Liu , Sci. Adv. 2022, 8, eade1136. 36475786 10.1126/sciadv.ade1136 PMC9728971

[5] S. B. Choi , A. M. Polter , P. Nemes , Anal. Chem. 2021, 94, 1637. 34964611 10.1021/acs.analchem.1c03826

[6] A. Obergrussberger , S. Friis , A. Brüggemann , N. Fertig , Expert Opin. Drug Discov. 2021, 16, 1. 32646308 10.1080/17460441.2020.1791079

[7] J. Gao , H. Zhang , P. Xiong , X. Yan , C. Liao , G. Jiang , TrAC Trends in Analytical Chemistry 2020, 133, 116082.

[8] K. Koos , G. Oláh , T. Balassa , N. Mihut , M. Rózsa , A. Ozsvár , E. Tasnadi , P. Barzó , N. Faragó , L. Puskás , Nat. Commun. 2021, 12, 936. 33568670 10.1038/s41467-021-21291-4 PMC7875980

[9] C. C. Petersen , Neuron 2017, 95, 1266. 28910617 10.1016/j.neuron.2017.06.049

[10] C. Liu , X. Cai , A. Ritzau‐Jost , P. F. Kramer , Y. Li , Z. M. Khaliq , S. Hallermann , P. S. Kaeser , Science 2022, 375, 1378. 35324301 10.1126/science.abn0532 PMC9081985

[11] J. Montnach , L. A. Blömer , L. Lopez , L. Filipis , H. Meudal , A. Lafoux , S. Nicolas , D. Chu , C. Caumes , R. Béroud , Nat. Commun. 2022, 13, 417. 35058427 10.1038/s41467-022-27974-w PMC8776733

[12] B. Juarez , M.‐S. Kong , Y. S. Jo , J. E. Elum , J. X. Yee , S. Ng‐Evans , M. Cline , A. C. Hunker , M. A. Quinlan , M. A. Baird , Sci. Adv. 2023, 9, eadg8869. 37566654 10.1126/sciadv.adg8869 PMC10421029

[13] V. H. Cornejo , N. Ofer , R. Yuste , Science 2022, 375, 82. 34762487 10.1126/science.abg0501 PMC8942082

[14] J. Y. Ryu , M. Y. Lee , J. H. Lee , B. H. Lee , K.‐S. Oh , Bioinformatics 2020, 36, 3049. 32022860 10.1093/bioinformatics/btaa075

[15] H. Kim , M. Park , I. Lee , H. Nam , Brief. Bioinform. 2022, 23, bbac211. 35709752 10.1093/bib/bbac211

[16] P. Rupprecht , S. Carta , A. Hoffmann , M. Echizen , A. Blot , A. C. Kwan , Y. Dan , S. B. Hofer , K. Kitamura , F. Helmchen , Nat. Neurosci. 2021, 24, 1324. 34341584 10.1038/s41593-021-00895-5 PMC7611618

[17] H. Wang , M. Xie , G. Rizzi , X. Li , K. Tan , M. Fussenegger , Adv. Sci. 2022, 9, 2102855. 10.1002/advs.202102855 PMC8895113 35040584

[18] N. Celik , F. O'Brien , S. Brennan , R. D. Rainbow , C. Dart , Y. Zheng , F. Coenen , R. Barrett‐Jolley , Commun. Biol. 2020, 3, 3. 31925311 10.1038/s42003-019-0729-3 PMC6946689

[19] M. Richter‐Laskowska , P. Trybek , P. Bednarczyk , A. Wawrzkiewicz‐Jałowiecka , Int. J. Mol. Sci. 2021, 22, 840. 33467711 10.3390/ijms22020840 PMC7831025

[20] M. Mortensen , T. G. Smart , Nat. Protoc. 2007, 2, 2826. 18007618 10.1038/nprot.2007.403

[21] J. Lv , X. Xiao , M. Bi , T. Tang , D. Kong , M. Diao , Q. Jiao , X. Chen , C. Yan , X. Du , Ageing Res. Rev. 2022, 80, 101676. 35724860 10.1016/j.arr.2022.101676

[22] G. Natale , V. Calabrese , G. Marino , F. Campanelli , F. Urciuolo , A. de Iure , V. Ghiglieri , P. Calabresi , M. Bossola , B. Picconi , Cell Death Discov. 2021, 7, 295. 34657122 10.1038/s41420-021-00685-9 PMC8520534

[23] J. Xu , W. Du , Y. Zhao , K. Lim , L. Lu , C. Zhang , L. Li , Acta Pharm. Sin. B 2022, 12, 2778. 35755284 10.1016/j.apsb.2022.03.001 PMC9214044

[24] J. L. Cummings , T. Morstorf , K. Zhong , Alzheimer's Res. Ther. 2014, 6, 1. 25024750 10.1186/alzrt269 PMC4095696

[25] H. Li , X. Sun , W. Cui , M. Xu , J. Dong , B. E. Ekundayo , D. Ni , Z. Rao , L. Guo , H. Stahlberg , S. Yuan , H. Vogel , Nat. Biotechnol. 2024, 42, 229. 38361054 10.1038/s41587-023-01987-2

[26] N. L. Harrison , G. W. Abbott , C. McClenaghan , C. G. Nichols , D. Cabrera‐Garcia , Nat. Chem. Biol. 2023, 19, 1303. 37798356 10.1038/s41589-023-01423-1

[27] H. Qian , X. Kang , J. Hu , D. Zhang , Z. Liang , F. Meng , X. Zhang , Y. Xue , R. Maimon , S. F. Dowdy , Nature 2020, 582, 550. 32581380 10.1038/s41586-020-2388-4 PMC7521455

[28] A. Porro , A. Saponaro , R. Castelli , B. Introini , A. Hafez Alkotob , G. Ranjbari , U. Enke , J. Kusch , K. Benndorf , B. Santoro , Nat. Commun. 2024, 15, 843. 38287019 10.1038/s41467-024-45136-y PMC10825183

[29] J. Wie , Z. Liu , H. Song , T. F. Tropea , L. Yang , H. Wang , Y. Liang , C. Cang , K. Aranda , J. Lohmann , Nature 2021, 591, 431. 33505021 10.1038/s41586-021-03185-z PMC7979525

[30] H. Liu , X. Xu , E. Li , S. Zhang , X. Li , IEEE Transactions on Neural Networks and Learning Systems 2021, 34, 2831. 10.1109/TNNLS.2021.3109898 34520369

[31] F. Scala , D. Kobak , M. Bernabucci , Y. Bernaerts , C. R. Cadwell , J. R. Castro , L. Hartmanis , X. Jiang , S. Laturnus , E. Miranda , Nature 2021, 598, 144. 33184512 10.1038/s41586-020-2907-3 PMC8113357

[32] J. Shin , L. Kovacheva , D. Thomas , S. Stojanovic , C. J. Knowlton , J. Mankel , J. Boehm , N. Farassat , C. Paladini , J. Striessnig , Sci. Adv. 2022, 8, eabm4560. 35675413 10.1126/sciadv.abm4560 PMC9177074

[33] N. Ju , Y. Li , F. Liu , H. Jiang , S. L. Macknik , S. Martinez‐Conde , S. Tang , Nat. Commun. 2020, 11, 697. 32019929 10.1038/s41467-020-14501-y PMC7000673

[34] X. Wang , X. Wang , W. Liu , Z. Chang , T. Kärkkäinen , F. Cong , Neurocomputing 2021, 459, 212.

[35] M. Fu , F. Hou , Front. Physiol. 2021, 12, 628502. 33746774 10.3389/fphys.2021.628502 PMC7965953

[36] A. Vaswani , N. Shazeer , N. Parmar , J. Uszkoreit , L. Jones , A. N. Gomez , Ł. Kaiser , I. Polosukhin , Adv. Neural Inf. Process. Syst. 2017, 30.

[37] D. Xie , K. Xiong , X. Su , G. Wang , L. Wang , Q. Zou , C. Zhang , Y. Cao , Y. Liu , Y.‐H. Chen , Cell Discov. 2022, 8, 76. 35918317 10.1038/s41421-022-00429-8 PMC9345967

[38] M. R. Wilcox , A. Nigam , N. G. Glasgow , C. Narangoda , M. B. Phillips , D. S. Patel , S. Mesbahi‐Vasey , A. L. Turcu , S. Vázquez , M. G. Kurnikova , Nat. Commun. 2022, 13, 4114. 35840593 10.1038/s41467-022-31817-z PMC9287434

[39] S. Zhang , P. Sun , K. Lin , F. H. L. Chan , Q. Gao , W. F. Lau , V. A. Roy , H. Zhang , K. W. C. Lai , Z. Huang , Adv. Sci. 2019, 6, 1901822. 10.1002/advs.201901822 PMC6918115 31871862

[40] L. Salkoff , R. Wyman , Nature 1981, 293, 228. 6268986 10.1038/293228a0

[41] A. I. Fernández‐Mariño , X.‐F. Tan , C. Bae , K. Huffer , J. Jiang , K. J. Swartz , Nature 2023, 622, 410. 37758949 10.1038/s41586-023-06582-8 PMC10567553

[42] M. Lipinsky , W. S. Tobelaim , A. Peretz , L. Simhaev , A. Yeheskel , D. Yakubovich , G. Lebel , Y. Paas , J. A. Hirsch , B. Attali , Sci. Adv. 2020, 6, eabd6922. 33355140 10.1126/sciadv.abd6922 PMC11206195

[43] J. Zhou , A. Jeron , B. London , X. Han , G. Koren , Circ. Res. 1998, 83, 806. 9776727 10.1161/01.res.83.8.806

[44] L. Hong , M. M. Pathak , I. H. Kim , D. Ta , F. Tombola , Neuron 2013, 77, 274. 23352164 10.1016/j.neuron.2012.11.013 PMC3559007

[45] S. Fenske , K. Hennis , R. D. Rötzer , V. F. Brox , E. Becirovic , A. Scharr , C. Gruner , T. Ziegler , V. Mehlfeld , J. Brennan , Nat. Commun. 2020, 11, 5555. 33144559 10.1038/s41467-020-19304-9 PMC7641277

[46] P. K. Tari , C. G. Parsons , G. L. Collingridge , G. Rammes , Neuropharmacology 2024, 244, 109737. 37832633 10.1016/j.neuropharm.2023.109737

[47] M. R. Wilson , S. Satapathy , M. Vendruscolo , Nat. Rev. Neurol. 2023, 19, 235. 36828943 10.1038/s41582-023-00786-2

[48] D. W. Sirkis , J. S. Yokoyama , Science 2023, 381, 1156. 37708264 10.1126/science.adk2009

[49] S. R. Williams , C. Wozny , Nat. Commun. 2011, 2, 242. 21407208 10.1038/ncomms1225 PMC3072097

[50] S. R. Williams , S. J. Mitchell , Nat. Neurosci. 2008, 11, 790. 18552844 10.1038/nn.2137

[51] Y. Han , J. Yang , Y. Li , Y. Chen , H. Ren , R. Ding , W. Qian , K. Ren , B. Xie , M. Deng , Sci. Adv. 2023, 9, eadi4208. 37992174 10.1126/sciadv.adi4208 PMC10664999

[52] G. Dai , T. K. Aman , F. DiMaio , W. N. Zagotta , Nat. Commun. 2021, 12, 2802. 33990563 10.1038/s41467-021-23062-7 PMC8121817

[53] P. Geurts , D. Ernst , L. Wehenkel , Mach. Learn. 2006, 63, 3.

[54] L. Breiman , Mach. Learn. 2001, 45, 5.

[55] J. R. Quinlan , Mach. Learn. 1986, 1, 81.

[56] G. Ke , Q. Meng , T. Finley , T. Wang , W. Chen , W. Ma , Q. Ye , T.‐Y. Liu , Adv. Neural Inf. Process. Syst. 2017, 30.

[57] T. Cover , P. Hart , IEEE transactions on information theory 1967, 13, 21.

[58] T. N. Sainath , O. Vinyals , A. Senior , H. Sak , in 2015 IEEE International Conference on Acoustics, Speech and Signal Processing (ICASSP), IEEE, 2015, pp. 4580‐4584.

[59] R. Caruana , Mach. Learn. 1997, 28, 41.

[60] R. McBeath , D. M. Pirone , C. M. Nelson , K. Bhadriraju , C. S. Chen , Dev. Cell 2004, 6, 483. 15068789 10.1016/s1534-5807(04)00075-9

[61] Y. Hou , W. Xie , L. Yu , L. C. Camacho , C. Nie , M. Zhang , R. Haag , Q. Wei , Small 2020, 16, 1905422. 10.1002/smll.201905422 32064782

[62] N. P. Nguyen , B. B. Laird , J. Phys. Chem. A 2023, 127, 9831. 37938899 10.1021/acs.jpca.3c04955

[63] M. Ashrafuzzaman , Membranes 2021, 11, 672.34564489

[64] C.‐k. Oh , T. Nakamura , N. Beutler , X. Zhang , J. Piña‐Crespo , M. Talantova , S. Ghatak , D. Trudler , L. N. Carnevale , S. R. McKercher , Nat. Chem. Biol. 2023, 19, 275. 36175661 10.1038/s41589-022-01149-6 PMC10127945

[65] C. M. Noviello , J. Kreye , J. Teng , H. Prüss , R. E. Hibbs , Cell 2022, 185, 2469. 35803245 10.1016/j.cell.2022.06.025 PMC9394431

[66] Z. Chen , A. Mondal , F. Abderemane‐Ali , S. Jang , S. Niranjan , J. L. Montaño , B. W. Zaro , D. L. Minor Jr. , Nature 2023, 619, 410. 37196677 10.1038/s41586-023-06175-5 PMC10896479

[67] H. Zhang , M. Lei , Y. Zhang , H. Li , Z. He , S. Xie , L. Zhu , S. Wang , J. Liu , Y. Li , Sci. Adv. 2024, 10, eadi7024. 38758791 10.1126/sciadv.adi7024 PMC11100570

[68] Y.‐C. Lin , M. Boone , L. Meuris , I. Lemmens , N. Van Roy , A. Soete , J. Reumers , M. Moisse , S. Plaisance , R. Drmanac , Nat. Commun. 2014, 5, 4767. 25182477 10.1038/ncomms5767 PMC4166678

[69] K. Burleigh , J. H. Maltbaek , S. Cambier , R. Green , M. Gale Jr , R. C. James , D. B. Stetson , Sci. Immunol. 2020, 5, eaba4219. 31980485 10.1126/sciimmunol.aba4219 PMC7081723

[70] J. Broche , A. R. Köhler , F. Kühnel , B. Osteresch , T. T. Chandrasekaran , S. Adam , J. Brockmeyer , A. Jeltsch , Commun. Biol. 2023, 6, 138. 36732350 10.1038/s42003-023-04466-1 PMC9895073

[71] M. Schewe , H. Sun , Ü. Mert , A. Mackenzie , A. C. Pike , F. Schulz , C. Constantin , K. S. Vowinkel , L. J. Conrad , A. K. Kiper , Science 2019, 363, 875. 30792303 10.1126/science.aav0569 PMC6982535

[72] J. She , J. Guo , Q. Chen , W. Zeng , Y. Jiang , X.‐c. Bai , Nature 2018, 556, 130. 29562233 10.1038/nature26139 PMC5886804

[73] M. Aarts , K. Iihara , W.‐L. Wei , Z.‐G. Xiong , M. Arundine , W. Cerwinski , J. F. MacDonald , M. Tymianski , Cell 2003, 115, 863. 14697204 10.1016/s0092-8674(03)01017-1

[74] J. P. Yuan , W. Zeng , G. N. Huang , P. F. Worley , S. Muallem , Nat. Cell Biol. 2007, 9, 636. 17486119 10.1038/ncb1590 PMC2699187

[75] Z. Ni , P. Qin , H. Liu , J. Chen , S. Cai , W. Tang , H. Xiao , C. Wang , G. Qu , C. Lin , ACS Nano 2023, 17, 20611. 37796740 10.1021/acsnano.3c07663 PMC10604094

[76] P. Sun , G. Qu , Q. Hu , Y. Ma , H. Liu , Z.‐X. Xu , Z. Huang , ACS Appl. Energy Mater. 2022, 5, 3568.

[77] J. Wu , X. Cui , P. C. Ke , M. Mortimer , X. Wang , L. Bao , C. Chen , Nano Today 2021, 41, 101328.

[78] Z. Zhang , Z. Liu , P. Wu , X. Guo , X. Luo , Y. Yang , J. Chen , Y. Tian , Adv. Sci. 2023, 10, 2301004. 10.1002/advs.202301004 PMC10582456 37635166

[79] J. Tromp , D. Bauer , B. L. Claggett , M. Frost , M. B. Iversen , N. Prasad , M. C. Petrie , M. G. Larson , J. A. Ezekowitz , S. D. Solomon , Nat. Commun. 2022, 13, 6776. 36351912 10.1038/s41467-022-34245-1 PMC9646849

[80] C. Xie , X.‐X. Zhuang , Z. Niu , R. Ai , S. Lautrup , S. Zheng , Y. Jiang , R. Han , T. S. Gupta , S. Cao , Nat. Biomed. Eng. 2022, 6, 76. 34992270 10.1038/s41551-021-00819-5 PMC8782726

[81] C. Han , W. Que , S. Wang , J. Zhang , J. Zhao , L. Shi , Expert Syst. Appl. 2022, 199, 117187.

[82] S. B. Kodandaramaiah , G. T. Franzesi , B. Y. Chow , E. S. Boyden , C. R. Forest , Nat. Methods 2012, 9, 585. 22561988 10.1038/nmeth.1993 PMC3427788

[83] F. Seibertz , M. Rapedius , F. E. Fakuade , P. Tomsits , A. Liutkute , L. Cyganek , N. Becker , R. Majumder , S. Clauß , N. Fertig , Commun. Biol. 2022, 5, 969. 36109584 10.1038/s42003-022-03871-2 PMC9477872

[84] S. Yang , K. W. C. Lai , J. Micro Bio Robot 2022, 18, 75.

[85] E. F. Harkin , M. B. Lynn , A. Payeur , J.‐F. Boucher , L. Caya‐Bissonnette , D. Cyr , C. Stewart , A. Longtin , R. Naud , J.‐C. Béïque , Elife 2023, 12, e72951. 36655738 10.7554/eLife.72951 PMC9977298

[86] V. B. Kasaragod , M. Mortensen , S. W. Hardwick , A. A. Wahid , V. Dorovykh , D. Y. Chirgadze , T. G. Smart , P. S. Miller , Nature 2022, 602, 529. 35140402 10.1038/s41586-022-04402-z PMC8850191

[87] A. Tzilivaki , J. J. Tukker , N. Maier , P. Poirazi , R. P. Sammons , D. Schmitz , Neuron 2023, 111, 3154. 37467748 10.1016/j.neuron.2023.06.016 PMC10593603

[88] H. Ye , B. Feng , C. Wang , K. Saito , Y. Yang , L. Ibrahimi , S. Schaul , N. Patel , L. Saenz , P. Luo , Sci. Adv. 2022, 8, eabk0185. 35044814 10.1126/sciadv.abk0185 PMC8769556

[89] Z. Liu , J. Zhou , Y. Li , F. Hu , Y. Lu , M. Ma , Q. Feng , J.‐e. Zhang , D. Wang , J. Zeng , Neuron 2014, 81, 1360. 24656254 10.1016/j.neuron.2014.02.010 PMC4411946

