# Supplementary material for: Deep Learning‐Based Ion Channel Kinetics Analysis for Automated Patch Clamp Recording
Source: Adv Sci (Weinh). 2024 Dec 31;12(12):2404166. doi: 10.1002/advs.202404166 (PMC12083860; doi:10.1002/advs.202404166)
Supplement: Supplementary file 1 — Supporting Information [file ADVS-12-2404166-s001.docx]

Supplementary

S1. Whole-cell Voltage Protocols

For muti-classification training, four protocols were used to capture the six categories of representative ion channel kinetics (Figure S1).

**Protocol I**: Due to different physiological conditions, patch clamp recordings using this protocol can result in three categories of recordings: Category I, II, and V. The protocol was designed to capture the typical activity of multiple ion channels as Category I, particularly fast inactivation and slow inactivation potassium channels.^[1]^ It also observes the presence of fast inactivation activity and the absence of non-inactivation activity (Category II).^[2]^ In cells lacking stability, the voltage dependence and selectivity of ion channels led to a state of disorder (Category V).

**Protocol II**: This protocol demonstrated the reduced activity of fast-inactivation potassium channels under low thermal condition (15℃), as Category III.^[3, 4]^ This protocol can also produce recordings in Categories I, II, and V. For example, with both partial failed fast inactivation and non-inactivation channels, the recordings can be similar to Category I but with different ion channel kinetics.

**Protocol III**: Slow activation/inactivation was indicated by the protocols, such as the voltage-gated potassium channels K_v_7.^[5]^ For normal cells, this protocol will produce recordings in Category I or III.

**Protocol IV**: The activities of hyperpolarization-activated channels were captured using negative activation voltages.^[6]^ Correspondingly, this protocol may produce recordings in Category II, III and IV in cells with HCN channel failure.

For anomaly detection model training, Protocol I and IV were used because they obtained all different types of signals. Due to anomalous ion channel activities, the recordings obtained using Protocol I were classified into Anomaly Signal I (aberrant transient recording), II (invalid recording), and III (low signal-to-noise ratio recording). Inconsistent hyperpolarization-activated channel activity in Anomaly Signal IV was captured using Protocol IV. The colored lines represent the various voltage stimuli. For Anomaly Signal I, II, and III, the blue, orange, green, red, and purple lines correspond to voltage stimuli at 100 mV, 80 mV, 60 mV, 40 mV, and 20 mV, respectively. For Anomaly Signal IV, the blue, orange, green, red, and purple lines correspond to voltage stimuli at -120 mV, -110 mV, -100 mV, -90 mV, and -80 mV, respectively.


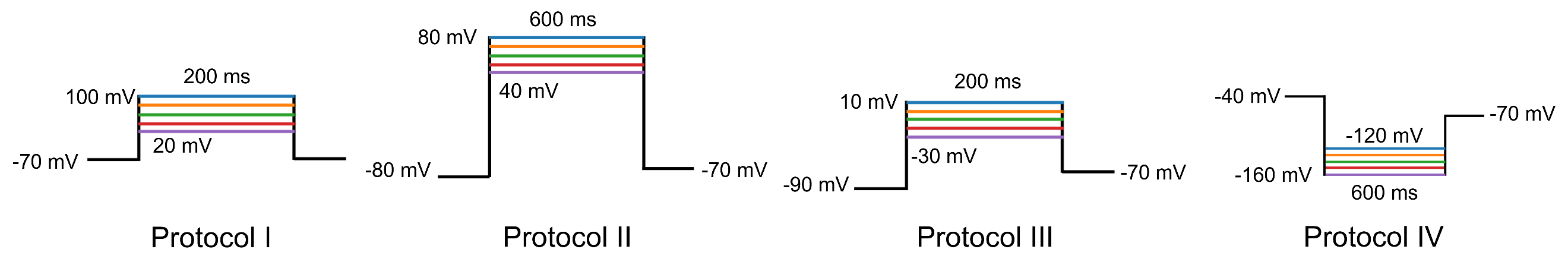


**Figure S1.** Whole-cell voltage-clamp protocols for obtaining various ion channel kinetics dataset.

For drug screening applications, Protocols V and VI were employed to investigate the kinetics of potassium and sodium ion channels, each following distinct protocols. They produced recordings in all six categories. In the nanomatrix-induced cell differentiation experiments, Protocols VII and VIII were utilized to assess potassium and sodium ion channel activities, respectively. In total, recordings from four categories were obtained to characterize the neurophysiological functions and electrophysiological properties.


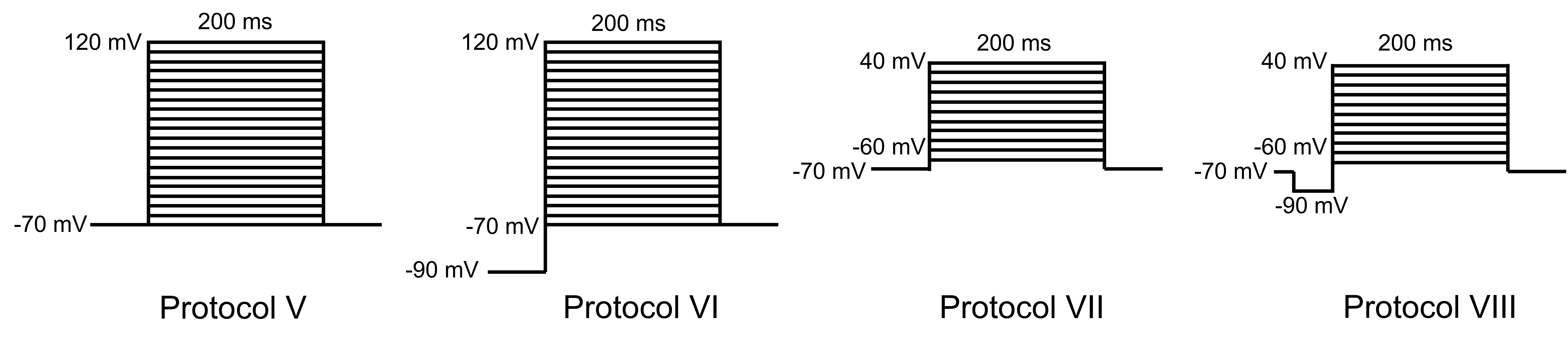


**Figure S2.** Whole-cell voltage-clamp protocols for drug screening and cell differentiation.

S2. Further Examples of Applying the Artificial Intelligence Classification Model on GABA Interneurons and 5-HT Neurons

The artificial intelligence framework further demonstrated its generalizability and reproducibility by analyzing the ion channel kinetics of γ-Aminobutyric acid (GABA) interneurons and serotonin (5-HT) neurons. The whole-cell recordings come from two commonly used public datasets obtained from other research groups.^[7, 8]^ In the practical applications, the artificial intelligence framework classified the A-type potassium activity of GABA interneurons and the fast inactivation activity of 5-HT neurons with 100% and 93.33% accuracy, respectively.

Applying the deep learning model on GABA interneurons, the whole-cell recordings are classified into three Category with 100% (13/13) accuracy (Figure S3a). GABA interneurons are widely distributed throughout the brain and spinal cord, regulating neuronal excitability and maintaining the balance between excitation and inhibition within neural circuits.^[9, 10]^ Typically, GABA interneurons exhibit A-type potassium channel activity. A-type potassium channel is the primary ion channel involved in the function of GABA interneurons for shaping the electrical behavior. The typical transient outward potassium currents of A-type channels are shown in Figure S3b. In commonly studies, 4-aminopyridine and tetraethylammonium ions are considered to inhibit this channel, as illustrated in Figure S3c. By classifying the recordings and evaluating the Category II and Category III, the functional and physiological status of interneurons will be accessed. For instance, the activation of endocannabinoid receptors reduces fast inactivation potassium currents. This pathology arises from the decreased strength of excitatory inputs to GABA interneurons where the response currents are consistent with the ion activity in Figure S3d.


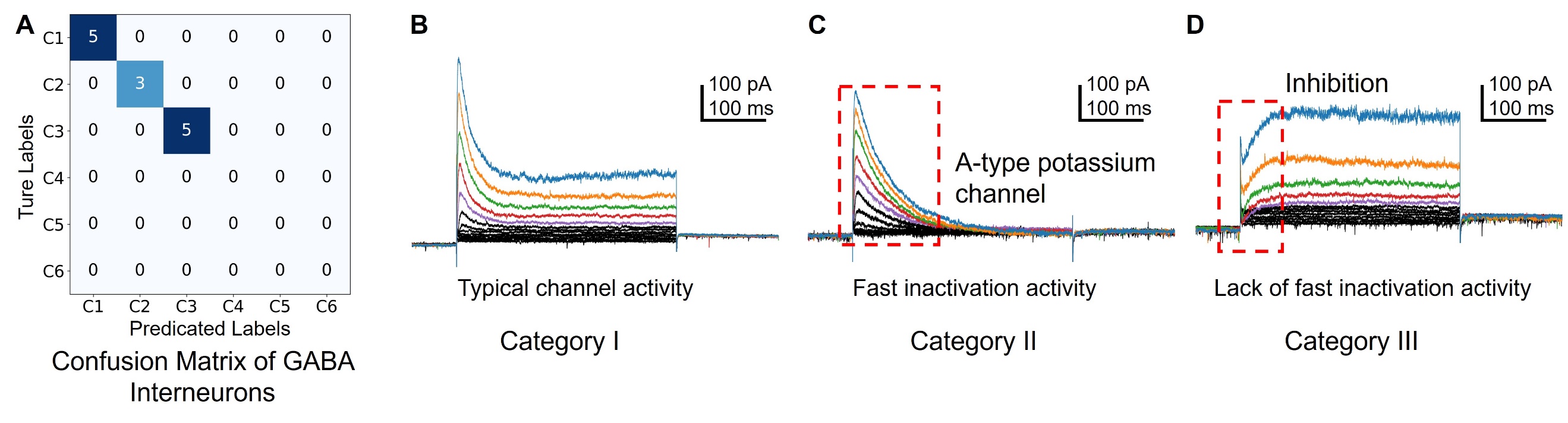


**Figure S3.** Characterization of GABA interneurons. (a) Classification of A-type potassium channels in GABA interneurons. (b) Representative whole-cell recording of GABA interneurons. (c) Response currents of A-type channels. (d) Response currents when A-type channels are inhibited.

In classifying fast inactivation potassium channel activity in 5-HT neurons, the artificial intelligence framework identified three categories, as shown in Figure S4a. By comparing the model-generated confusion matrix with expert-annotated results, the deep learning model achieved an accuracy of 93.33% (14/15). Axons of 5-HT neurons project to various regions of the brain and spinal cord, significantly influencing mood regulation. Alterations in serotonin signaling are linked to psychiatric disorders such as depression, anxiety, and schizophrenia.^[11, 12]^ For signaling transmission, the fast activation potassium channel is activated and inactivated within a short period, introducing a transient outward current to offset depolarization (Figure S4b). This shapes the action potential and controls excitability, including desynchronizing neuronal populations and modulating the gain of transient responses. Dysfunction arises from two aspects: absence and partial activation of fast inactivation potassium channels. The absence of fast inactivation channels leads to complete non-functionality (Figure S4c). Due to the lack of outward potassium currents, inward sodium currents may be observed. Partial activation delays the onset of action potentials by increasing the threshold for action potential initiation, resulting in a reduction in firing frequency (Figure S4d).


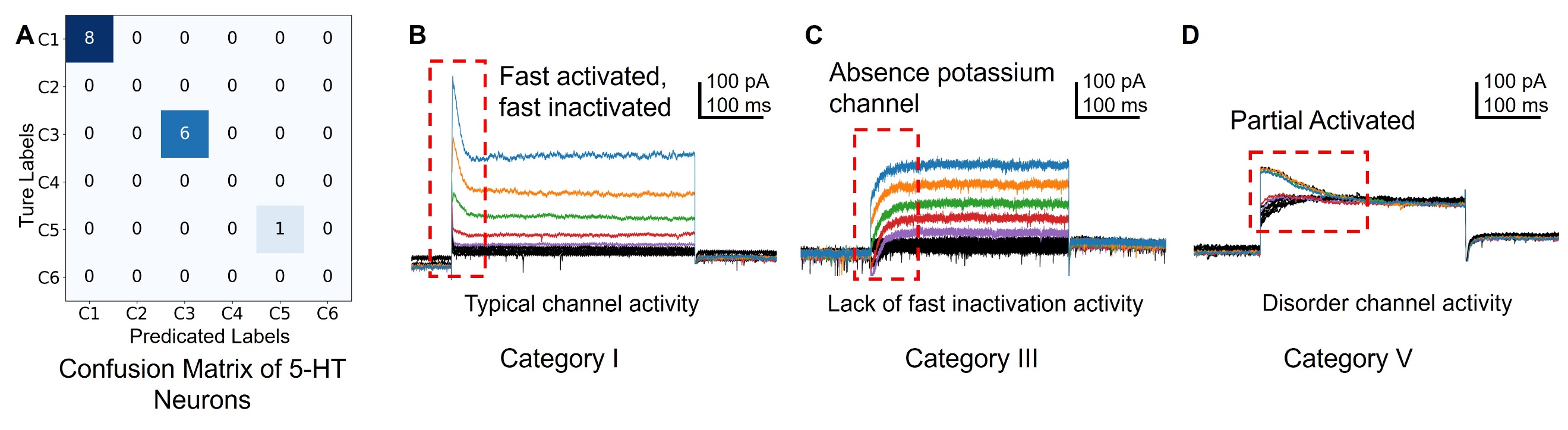


**Figure S4.** Effects of fast inactivation activity on action potentials. (a) Categories of fast inactivation potassium channel activity. (b) Fast activation fast inactivation potassium channels. (c) Lack of fast activation potassium currents and presence of tight inward sodium currents. (d) Disordered voltage dependence leading to erroneous ion channel activity.

S3. Validation Procedures in Model Training

The loss and accuracy over epochs during the development of the deep learning model are presented as Figure S5, including both training and validation stages. Both training and validation losses decrease rapidly during the initial epochs and gradually plateau at 0.025 and 0.013, respectively. The continuous decrease in training loss indicates that the model is learning and fitting the training data better over time. The validation loss also decreases and closely follows the training loss, suggesting that the model is generalizing well on the validation data. The validation accuracy increases rapidly during the initial epochs and gradually plateaus at 0.9898. The close match between validation accuracy and training accuracy suggests that the model is performing well without significant overfitting or underfitting. The confidence intervals of the validation estimates stabilize over time, indicating consistent performance.


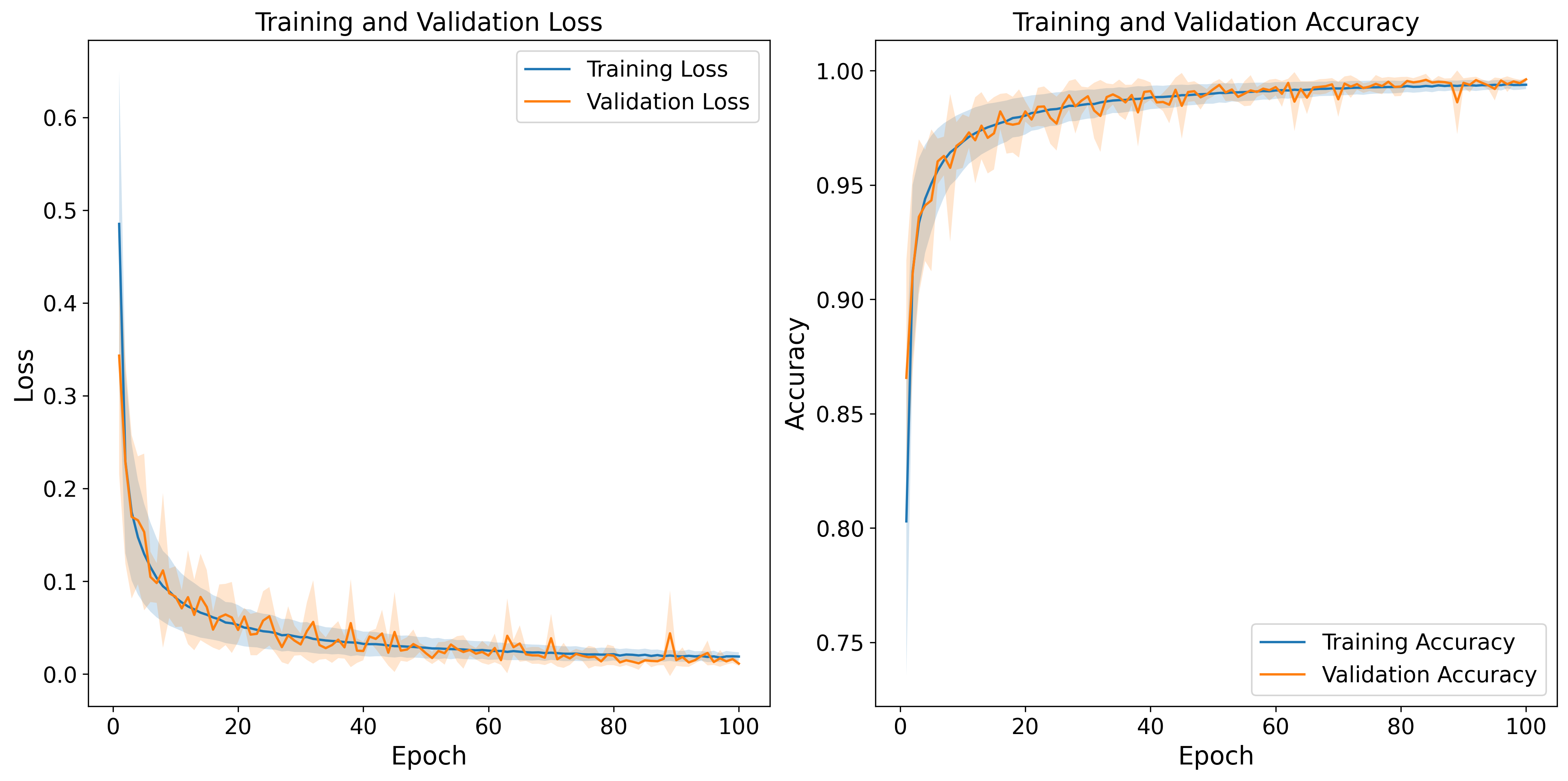


**Figure S5.** The loss and accuracy in training and validation over epochs.

References

[1] J. Huguenard, D. Coulter, D. Prince, *Journal of neurophysiology* **1991**, *66* (4), 1304.

[2] C.-C. Chen, C. Cang, S. Fenske, E. Butz, Y.-K. Chao, M. Biel, D. Ren, C. Wahl-Schott, C. Grimm, *Nature Protocols* **2017**, *12* (8), 1639.

[3] A. I. Fernández-Mariño, X.-F. Tan, C. Bae, K. Huffer, J. Jiang, K. J. Swartz, *Nature* **2023**, *622* (7982), 410.

[4] R. Ranjan, E. Logette, M. Marani, M. Herzog, V. Tâche, E. Scantamburlo, V. Buchillier, H. Markram, *Frontiers in Cellular Neuroscience* **2019**, *13*, 358.

[5] J. Johnston, I. D. Forsythe, C. Kopp‐Scheinpflug, *The Journal of physiology* **2010**, *588* (17), 3187.

[6] F. C. Roth, H. Hu, *Nature Communications* **2020**, *11* (1), 2248.

[7] E. F. Harkin, M. B. Lynn, A. Payeur, J.-F. Boucher, L. Caya-Bissonnette, D. Cyr, C. Stewart, A. Longtin, R. Naud, J.-C. Béïque, *Dryad* **2023**, DOI:10.5061/dryad.66t1g1k2w.

[8] E. F. Harkin, M. B. Lynn, A. Payeur, J.-F. Boucher, L. Caya-Bissonnette, D. Cyr, C. Stewart, A. Longtin, R. Naud, J.-C. Béïque, *Elife* **2023**, 12, e72951.

[9] V. B. Kasaragod, M. Mortensen, S. W. Hardwick, A. A. Wahid, V. Dorovykh, D. Y. Chirgadze, T. G. Smart, P. S. Miller, *Nature* **2022**, 602 (7897), 529.

[10] A. Tzilivaki, J. J. Tukker, N. Maier, P. Poirazi, R. P. Sammons, D. Schmitz, *Neuron* **2023**, 111 (20), 3154.

[11] H. Ye, B. Feng, C. Wang, K. Saito, Y. Yang, L. Ibrahimi, S. Schaul, N. Patel, L. Saenz, P. Luo, *Science advances* **2022**, 8 (3), eabk0185.

[12] Z. Liu, J. Zhou, Y. Li, F. Hu, Y. Lu, M. Ma, Q. Feng, J.-e. Zhang, D. Wang, J. Zeng, *Neuron* **2014**, 81 (6), 1360.
